# Dietary selection of metabolically distinct microorganisms drives hydrogen metabolism in ruminants

**DOI:** 10.1038/s41396-022-01294-9

**Published:** 2022-08-05

**Authors:** Qiu Shuang Li, Rong Wang, Zhi Yuan Ma, Xiu Min Zhang, Jin Zhen Jiao, Zhi Gang Zhang, Emilio M. Ungerfeld, Kang Le Yi, Bai Zhong Zhang, Liang Long, Yun Long, Ye Tao, Tao Huang, Chris Greening, Zhi Liang Tan, Min Wang

**Affiliations:** 1 Key Laboratory for Agro-Ecological Processes in Subtropical Region, Institute of Subtropical Agriculture, Chinese Academy of Sciences, Changsha, Hunan China; 2 University of Chinese Academy of Sciences, Beijing, China; 3 College of Pastoral Agriculture Science and Technology, Lanzhou University, Lanzhou, China; 4 State Key Laboratory for Conservation and Utilization of Bio-Resources in Yunnan, School of Life Sciences, Yunnan University, Kunming, Yunnan China; 5 Centro Regional de Investigación Carillanca, Instituto de Investigaciones Agropecuarias (INIA), Temuco, Chile; 6 Hunan Institute of Animal and Veterinary Science, Changsha, Hunan China; 7 Xiangxi Cattle Engineering Technology Center of Hunan Province, Huayuan, Hunan China; 8 Hunan De Nong Animal Husbandry Group Co. Ltd, Huayuan, Hunan China; 9 Shanghai BIOZERON Biotechnology Company Ltd, Shanghai, China; 10 Shanghai Institute of Nutrition and Health, Chinese Academy of Sciences, Shanghai, China; 11 Biomedicine Discovery Institute, Department of Microbiology, Monash University, Clayton, Australia

**Keywords:** Metagenomics, Metagenomics

## Abstract

Ruminants are important for global food security but emit the greenhouse gas methane. Rumen microorganisms break down complex carbohydrates to produce volatile fatty acids and molecular hydrogen. This hydrogen is mainly converted into methane by archaea, but can also be used by hydrogenotrophic acetogenic and respiratory bacteria to produce useful metabolites. A better mechanistic understanding is needed on how dietary carbohydrates influence hydrogen metabolism and methanogenesis. We profiled the composition, metabolic pathways, and activities of rumen microbiota in 24 beef cattle adapted to either fiber-rich or starch-rich diets. The fiber-rich diet selected for fibrolytic bacteria and methanogens resulting in increased fiber utilization, while the starch-rich diet selected for amylolytic bacteria and lactate utilizers, allowing the maintenance of a healthy rumen and decreasing methane production (*p* < 0.05). Furthermore, the fiber-rich diet enriched for hydrogenotrophic methanogens and acetogens leading to increased electron-bifurcating [FeFe]-hydrogenases, methanogenic [NiFe]- and [Fe]-hydrogenases and acetyl-CoA synthase, with lower dissolved hydrogen (42%, *p* < 0.001). In contrast, the starch-rich diet enriched for respiratory hydrogenotrophs with greater hydrogen-producing group B [FeFe]-hydrogenases and respiratory group 1d [NiFe]-hydrogenases. Parallel in vitro experiments showed that the fiber-rich selected microbiome enhanced acetate and butyrate production while decreasing methane production (*p* < 0.05), suggesting that the enriched hydrogenotrophic acetogens converted some hydrogen that would otherwise be used by methanogenesis. These insights into hydrogen metabolism and methanogenesis improve understanding of energy harvesting strategies, healthy rumen maintenance, and methane mitigation in ruminants.

## Introduction

Ruminants have evolved to harbor a complex rumen microbial ecosystem of bacteria, archaea, protozoa, and fungi, which convert complex plant-cell carbohydrates into volatile fatty acids (VFA) and microbial protein usable by the host animal [1, 2]. Molecular hydrogen (H_2_) is produced during carbohydrate fermentation by anaerobic bacteria, protists, and fungi and is primarily consumed by methane (CH_4_)-producing archaea in the rumen [3, 4]. This H_2_ is then consumed by a range of bacteria as an energy source and electron donor, which ensures fermentation remains thermodynamically favorable [5]. Hydrogen cycling is catalyzed by H_2_-producing and H_2_-consuming hydrogenases classified into [FeFe]-, [NiFe]-, and [Fe]-hydrogenases based on metal content of their H_2_-binding sites [6]. Hydrogenases are widespread in bacterial and archaeal genomes, reflecting the metabolic importance of H_2_ transactions in rumen fermentation [5, 7, 8]. Methane produced from enteric fermentation not only represents a loss of dietary energy but also contributes to global anthropogenic greenhouse gas emissions [9, 10]. Importantly, rumen bacteria also encode hydrogenases and terminal reductases for hydrogenotrophic growth, using electron acceptors such as CO_2_, fumarate, sulfate, and nitrate. Unlike methanogenesis, some of these alternative hydrogenotrophic pathways produce metabolites usable by the host animal, for example acetate through hydrogenotrophic acetogenesis [5]. It is therefore important to understand how hydrogenotrophic bacteria compete with methanogens for the rumen H_2_, as this knowledge may facilitate metabolic strategies to enhance energy harvesting with reduced CH_4_ production in ruminants.

The composition of dietary carbohydrates drives microbial composition and function, including H_2_ metabolism and methanogenesis [11, 12]. When fed cellulosic plant material, rumen microorganisms hydrolyze and ferment lignocellulose primarily through the acetate pathway releasing H_2_, resulting in high levels of CH_4_ production [13]. However, some herbivores such as kangaroos and yak produce less CH_4_ [14, 15]. Hydrogenotrophic acetogenic bacteria can convert H_2_ and CO_2_ into acetate *via* the Wood–Ljungdahl pathway [5]. Some wild animals in regions where forage quantity or quality is low may have evolved digestive systems with enhanced hydrogenotrophic acetogenesis to promote the efficiency of dietary energy extraction and utilization [16]. It remains to be determined whether diet quality alters the fate of H_2_ produced, and thus influences the ratio of hydrogenotrophic acetogenesis to methanogenesis and efficiency of energy harvesting in farmed ruminants.

Despite being widely used, starch-rich high-grain diets present risks for ruminant health. High starch intake stimulates rapid VFA and lactate production in non-adapted rumens, leading to a rapid and sometimes pronounced drop in rumen pH [17]. Even in animals adapted to grain, a prolonged acidic rumen, a condition termed subacute rumen acidosis [18], results in reduced feed intake, chronic inflammation, rumen papillae dysfunction, and other diseases [19]. Unlike acetate production, lactate production is not associated with H_2_ production and facilitates reoxidation of redox cofactors (e.g., NADH to NAD^+^) [20]. However, given lactate has a lower pKa than VFAs, its accumulation decreases rumen pH and hence rapid conversion of lactate to propionate and other VFAs by lactate-utilizing bacteria is required to maintain a normal rumen function. In order to promote a healthy rumen while keeping CH_4_ production at low levels, it is important to understand how the conversion of lactate to propionate is controlled.

Altogether, previous studies suggest that rumen microbiota exhibits distinct metabolic strategies to adapt to fiber-rich or starch-rich diets. However, we lack a detailed mechanistic understanding of the microbiota and pathways of H_2_ metabolism affected by dietary shifts. To address this knowledge gap, we conducted one in vivo and two in vitro experiments, and developed a rumen model of fiber-rich and starch-rich selected microbiomes. We observed that feeding fiber-rich versus starch-rich diets selected for distinct rumen microbiome compositions, capabilities, and activities. Using metagenomic profiling, we found that microbiota resulting from feeding these two diets exhibited distinct carbohydrate and H_2_ metabolic pathways, with differences in downstream methanogenesis and VFA production pathways. Results also suggest a potential role in the rumen of hydrogenotrophic acetogenesis as a H_2_ sink other than methanogenesis with the fiber-rich dietary treatment, which might compete for H_2_ available for methanogenesis and produce extra energy available for the host animal. This study emphasizes that dietary interventions modulate rumen microbiota composition and in turn carbohydrate degradation, H_2_ metabolism, and methanogenesis.

## Material and methods

### Animals and experimental design

All animals involved in the experiment were cared for in accordance with the Animal Care and Use Guidelines of the Animal Care Committee, Institute of Subtropical Agriculture, the Chinese Academy of Sciences, Changsha, China, with all animal experimental procedures approved by the committee (approval number ISA-W-201901).

**Fig. 1:**
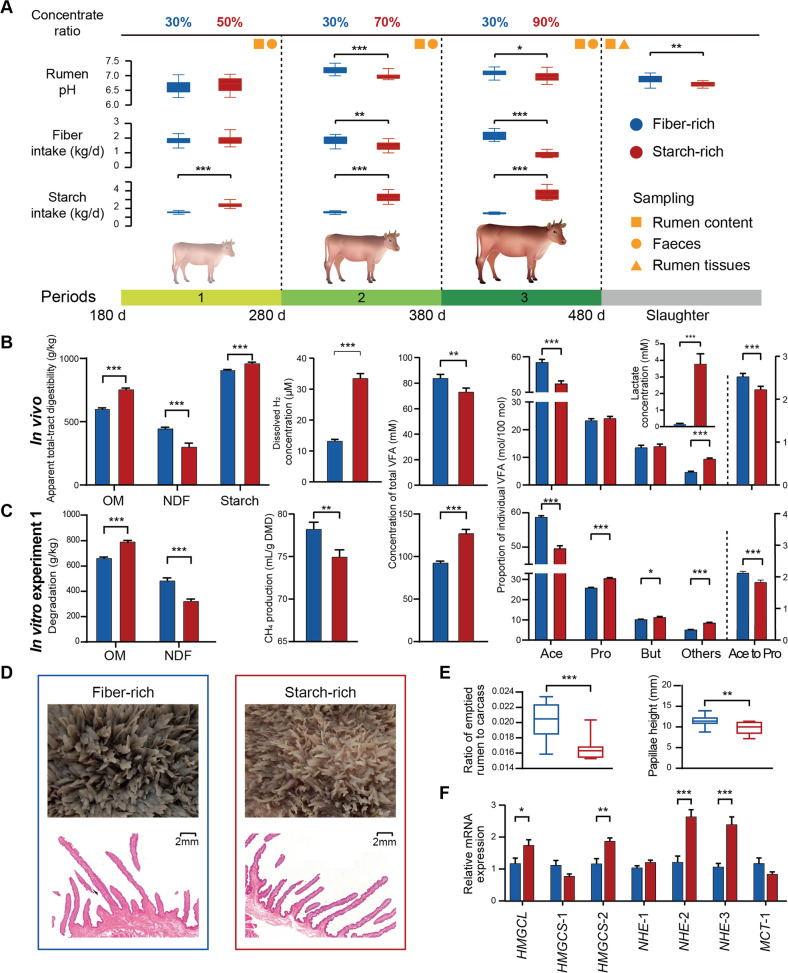
Fiber-rich and starch-rich treatment exhibits distinct microbial activity, papillae morphology, and function in bovine rumen. **A** Overview of daily fiber and starch intake (kg/d) and rumen pH over three experimental periods; **B** feed digestibility, dissolved hydrogen (H_2_), lactate and volatile fatty acids profile in vivo; **C** feed degradation, methane (CH_4_) production and volatile fatty acids profile in vitro rumen experiment 1; **D** inspection of morphological changes in rumen papillae; **E** ratio of emptied rumen to carcass and papillae height; **F** q-PCR results of genes related to volatile fatty acids absorption and intracellular pH regulation in ruminal epithelium. OM organic matter, NDF neutral detergent fiber, VFA volatile fatty acid, Ace acetate, Pro propionate, But butyrate; others, valerate, isovalerate and isobutyrate; HMGCL 3-hydroxy-3-methylglutaryl-CoA lyase; HMGCS-1 3-hydroxy-3-methylglutaryl-CoA synthase, isoform 1, HMGCS-2 3-hydroxy-3-methylglutaryl-CoA synthase, isoform 2, NHE-1 Na^+^/H^+^ exchanger 1, NHE-2 Na^+^/H^+^ exchanger 2, NHE-3 Na^+^/H^+^ exchanger 3, MCT-1 monocarboxylate transporter, isoform 1. Data with error bars are expressed as mean ± standard error. *p < 0.05, **p < 0.01, ***p < 0.001, *n* = 12/group.

A total of 24 local breed (Xiangxi) beef cattle (initial body weight 147 ± 9.8 kg) were randomly assigned to one of two dietary treatments that lasted for 300 d with three periods (Fig. 1A). The fiber-rich diet was kept constant in all three experimental periods and was formulated to have a forage neutral detergent fiber (NDF) content greater than 40% dry matter (DM) basis by including corn stover and rice straw. Three starch-rich diets with increasing starch content were formulated by gradually replacing one third of corn stover with maize meal (DM basis) in each experimental period, until reaching 90% of concentrate (DM basis) in period 3 (Supplemental Table S1). All the cattle were fed twice daily (7 a.m. and 5 p.m.) and had free access to drinking water.

### Samples collection

Full details are provided in Supplementary Materials and Methods. Briefly, nutrient digestibility was measured between days 93 and 98 of each of the three periods. Rumen contents were collected at 0, 2.5 and 6 h after the morning feeding on days 99 and 100 of each of the three periods. Rumen pH and dissolved hydrogen concentrations were immediately measured after collecting rumen contents, while other subsamples were frozen for DNA extraction and fermentation product measurements. All the cattle were slaughtered at the end of experiment after fasting for 12 h, and rumen contents and epithelial samples were collected.

### In vitro ruminal fermentation

Detailed information is provided in Supplementary Materials and Methods. The present study included two batch culture ruminal in vitro experiments conducted at final experimental period. In vitro experiment 1 compared ruminal fermentation between fiber-rich and starch-rich diets as incubation substrates, with each substrate being incubated with the rumen inoculum from animals fed the corresponding diet. In vitro experiment 2 compared the activity of fiber-rich and starch-rich selected microbiomes by inoculating rice straw with rumen inoculum from animals fed the fiber-rich and starch-rich diets, respectively. In vitro ruminal fermentation was performed according to the procedure of Wang et al. [21].

### Sample analysis

All detailed analysis procedures are provided in Supplementary Materials and methods. Feed and feces composition were analyzed using the methods of the Association of Official Analytical Chemists [22], Van Soest et al. [23] and Karthner and Theurer [24]. Dissolved H_2_ and CH_4_ were extracted from the liquid phase of rumen contents into the gas phase, measured using a gas chromatograph (GC, Agilent 7890A, Agilent Inc., Palo Alto, CA), and concentration calculated using the equation of Wang et al. [25]. Individual volatile fatty acid (VFA) concentrations were analyzed by GC (Agilent 7890A, Agilent Inc., Palo Alto, CA) [25], while the concentration of lactate was analyzed by high-performance liquid chromatography (HPLC, Agilent LC1290, Agilent Inc., Palo Alto, CA) [26].

DNA was extracted from rumen samples using repeated bead beating and column filtration as described [27]. Total RNA of the ruminal epithelium was extracted from the tissue after removing the genomic DNA. Quantitative real-time PCR (q-PCR) was performed to measure relative gene expression of seven genes in ruminal epithelium (*HMGCL*, *HMGCS*-1, *HMGCS*-2, *NHE*-1, *NHE*-2, *NHE*-3, *MCT*-1), the expression of which were normalized to the reference genes (*GAPDH* and *β-actin*) using a 2^–ΔΔCt^ method [28]. Absolute quantification of specific microbial taxa was performed according to the procedures described by Ma et al. [29].

### Bioinformatics analyses

All bioinformatics software, parameters, and databases involved in the study are present in Supplementary Materials and Methods. Briefly, the V3 and V4 region was used for 16S rRNA gene sequencing, with all amplicon libraries preparation and sequencing performed on a MiSeq platform (Illumina, San Diego, CA, USA). A total of 1,238,536 high-quality reads were generated with an average of 51,606 ± 1737 reads per sample for assignment of amplicon sequence variants (ASVs) by DADA2. Taxonomy was annotated using SILVA (release 138, http://www.arb-silva.de). For shotgun metagenome sequencing, 1 μg of genomic DNA was sheared by a Covaris S220 Focused-ultrasonicator (Woburn, MA USA) and sequencing libraries were prepared with a length of approximately 350 bp (ranging from 300 to 400 bp). Sequencing of metagenomic libraries was performed on the HiSeq X platform (Illumina, San Diego, CA, USA) in paired-end 150 bp (PE150) mode. After quality control, assembly, prediction, and clustering into a non-redundant gene set, the annotations of the gene set were performed with KEGG, CAZy, HydDB, and NCBI-NR, and the gene abundance within total samples were calculated. Microbial genomes were further verified by using Hungate1000 collection, which includes virtually all the bacterial and archaeal species that have been cultivated from the rumen of a diverse group of animals [30]. The abundance of each genome was calculated by metawrap quant_bins module with default parameters [31].

### Statistical analysis

The metabolic data were analyzed by one-way analysis of variance (ANOVA) in SPSS 21.0 software (SPSS Inc., Chicago, IL). When sampling time was included, data were analyzed using a linear mixed model with treatment and treatment by sampling time interaction as fixed effects, and sampling time as a repeated measures variable. The microbial community composition data was analyzed using Wilcoxon rank-sum test in the JMP Pro software (JMP Pro version 13.2.1, SAS Institute Inc. SAS Institute, Cary, NC, USA). The genomic ranks of attributes were evaluated by Correlation, ReliefF, Symmetrical Uncert, and multi-cluster feature selection (MCFS) methods in software of Waikato Environment for Knowledge Analysis (WEKA) (version 3.8.4, Hamilton, New Zealand) [32], and further comprehensively analyzed by RobustRankAggreg (RRA) R package [33]. All *p* values were adjusted for False Discovery Rate (FDR) using the Benjamini-Hochberg method. Statistical significance was declared at *p* ≤ 0.05 and tendencies at 0.05 < *p* ≤ 0.10.

## Results

### Volatile fatty acid production and absorption is modulated by diet

Animals were adapted to a starch-rich diet by gradually increasing dietary concentrate content from 50 to 90% over the three 100-d experimental periods (Fig. 1A). With both diets, rumen structure and epithelial morphology were robust and rumen pH remained above 6.0 (Fig. 1A, D Additional Table S4), indicating a healthy rumen function throughout the entire experiment.

Cattle with high fiber intake exhibited greater fiber digestibility, while cattle adapted to the starch-rich diet had greater organic matter and starch digestibility but lower fiber digestibility in vivo and in the in vitro experiment 1 (Fig. 1B, C, Additional Table S4, *p* < 0.001). Cattle adapted to the starch-rich diet exhibited greater rates of carbohydrate degradation, leading to increased total VFA concentration in periods 1 and 2 in vivo (Additional Table S4), and in the in vitro experiment 1 (Fig. 1C, *p* < 0.001). The lower VFA concentration (*p* < 0.01) observed in vivo at period 3 with the starch-rich diet could have been the result of a greater increase in the rate of VFA absorption than production, as suggested by the increased copy number of some of the genes related to VFA absorption and intracellular pH regulation in the ruminal epithelium (Fig. 1F), namely the limiting enzyme in ketone formation 3-hydroxy-3-methylglutaryl-CoA lyase (*HMGCL*), 3-hydroxy-3-methylglutaryl-CoA synthase isoform 2 (*HMGCS*-2), Na^+^/H^+^ exchanger 2 (*NHE*-2), and Na^+^/H^+^ exchanger 3 (*NHE*-3) [34–36]. Changes in VFA absorption between diets are also suggested by the alternation of rumen organ structure, with a lower ratio of rumen organ to carcass and shorter rumen papilla in the starch-rich diet (Fig. 1D, E).

Acetate molar percentage and the acetate to propionate molar ratio were higher in cattle adapted to the fiber-rich diet (*p* < 0.001). Such differences in VFA profiles agree with the in vitro ruminal fermentation results (Fig. 1B, C, Additional Table S4). The starch-rich diet increased (*p* < 0.001) ruminal dissolved H_2_ (dH_2_, 2.5-fold higher) and reduced CH_4_ production (*p* = 0.003). Altogether, these results suggest that the two diets differentially influence VFA production, H_2_ metabolism, and methanogenesis, likely due to the promotion of distinct microbial activities.

### Different microbial taxa were enriched by the two diets

**Fig. 2:**
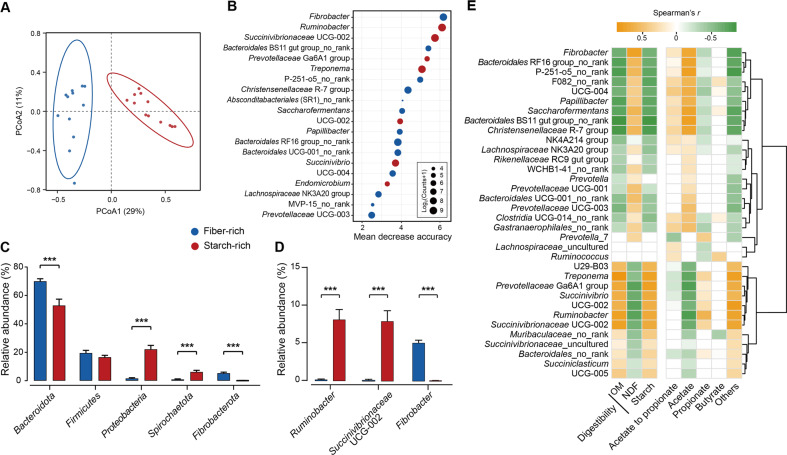
Fiber-rich and starch-rich treatments select distinct microbial communities. **A** principal coordinate analysis (PCoA) profile of ruminal bacterial community based on unweighted UniFrac dissimilarity matrix at the taxa (ASVs) level (PERMANOVA, *p* = 0.001, *R*^2^ = 0.19); **B** random forest analysis of rumen bacterial community. The *y*-axis, from top to bottom, displays the genera ranked by their relative importance based on mean decrease accuracy in the classification of groups; **C** relative abundance of the top five phyla; **D** relative abundance of genus *Fibrobacter*, *Ruminobacter*, and *Treponema*; **E** the relationships of feed digestibility and volatile fatty acids with bacterial community at genus level (average percent > 0.5%). No rank means there is no specific taxonomic information at the genus level. Only Spearman’s significance levels *p* < 0.05 are shown, yellow and blue indicate negative and positive correction respectively. OM organic matter; NDF neutral detergent fiber. Significance was tested using independent two-group Wilcoxon rank-sum tests. Data with error bars are expressed as mean ± standard error. **p < 0.01, ***p < 0.001, *n* = 12/group.

16S rRNA gene sequencing was used to examine rumen microbial community composition. Across the 24 samples, 3028 amplicon sequence variants (ASVs) were observed. Microbial composition clearly clustered by diet, based on unweighted UniFrac dissimilarity (Fig. 2A), indicating that carbohydrate type drove marked changes in the microbial community composition. In addition, the random forest was further applied to classify groups and selected the 20 most important bacterial genera ranked by mean decrease accuracy of classification of bacterial communities by diet (Fig. 2B, Additional File S1). Among them, *Ruminobacter and Succinivibronaceae* UCG-002 were the two most representative genera for the starch-rich diet, whilst *Fibrobacter* was the most representative genus in case of the fiber-rich diet. Furthermore, correlation network analysis of these representative bacterial genera revealed strong positive correlations within each treatment, and negative correlations between the treatments (*p* < 0.05, Spearman’s | *r *| > 0.5, Additional Fig. S3).

At the phylum level, *Bacteroidota* (*Bacteroidetes*) and *Fibrobacterota* (*Fibrobacteres*) were significantly enriched with the fiber-rich diet, whereas *Proteobacteria* and *Spirochaetota* (*Spirochaetes*) were more abundant with the starch-rich diet (Fig. 2B, *p* < 0.001). It should be noted that, due to the specificity of the primer pairs used (targeting the V3–V4 primer), *Spirochaetota* and archaea are potentially underrepresented in this analysis [37, 38]. At the genus level, *Fibrobacter* (37.3-fold higher), *Christensenellaceae* R-7 group (2.6-fold higher), and *Bacteroidales* RF16 group (4.0-fold higher) were enriched with the fiber-rich diet, while *Ruminobacter* (12.0-fold higher), *Treponema* (6.9-fold higher), and *Succinivibrionaceae* UCG-002 (21.8-fold higher) were enriched with the starch-rich diet (Fig. 2D, Additional Fig. S2, *p* < 0.001). Four of these genera were also among those with the highest discriminatory power based on the random forest analysis (Fig. 2B). In addition, *Fibrobacter*, *Bacteroidales* RF16 group, and *Christensenellaceae* R-7 group were positively correlated with acetate concentration and neutral detergent fiber (NDF) digestibility, while *Ruminobacter*, *Succinivibrionaceae* UCG-002, and *Treponema* were positively correlated with propionate concentration and starch digestibility (Fig. 2E). Differences in microbial composition are thus associated with distinct substrate preferences and degradation abilities.

### Distinct pathways of carbohydrate degradation are selected by two diets

**Fig. 3:**
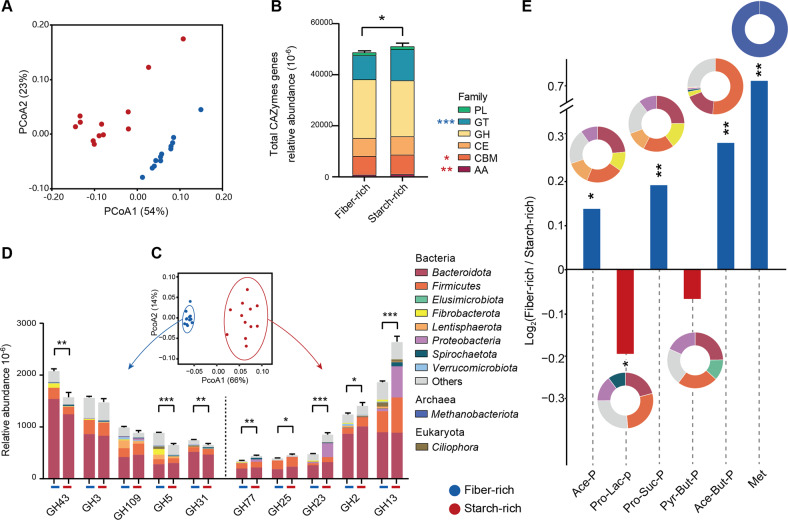
Fiber-rich and starch-rich treatment exhibits distinct genes of CAZymes and KEGG enzymes enriched in rumen microbiome. **A** PCoA profile of all KO genes (PERMANOVA, *P* < 0.001, *R*^2^ = 0.70); **B** relative abundance of total of CAZymes genes; **C** PCoA profile of the GH family (PERMANOVA, *P* < 0.001, *R*^2^ = 0.64); **D** gene abundance of the top five GH family enzymes, and their phylogenetic distribution enriched per phylum (AA auxiliary activity, CBM carbohydrate-binding module, CE carbohydrate esterase, GH glycoside hydrolase, GT glycosyltransferase, PL polysaccharide lyase); **E** acetate, butyrate, propionate and methanogenesis pathways of KEGG enzymes expressed as ratios of alignments of fiber-rich versus starch-rich treatment (log_2_ ratio); and pie charts show the phylogenetic distribution of the pathways enriched in each treatment at phylum. Ace-P acetate production pathway, Pyr-But-P pyruvate to butyrate production pathway, Ace-But-P acetate to butyrate production pathway, Pro-Lac-P propionate (lactate) pathway, Pro-Suc-P propionate (succinate) pathway, Met methanogenesis pathway. All KO genes in enriched pathways were assigned to the identified phylum. Details of all KO genes together with identified genus and pathways are in additional files S4 and S5. Significance was tested using independent two-group Wilcoxon rank-sum tests. Data with error bars are expressed as mean ± standard error. Asterisks denote significant adjusted *p* values: **p < 0.01, ***p < 0.001, *n* = 12/group.

After removing the reads assigned to the host, we obtained 503 Gb of paired-end sequencing data, which averaged 21.0 Gb (ranging from 17.4 to 29.1 Gb) per sample. We first investigated the metabolic capacities of the rumen microbiomes by using the Kyoto Encyclopedia of Genes and Genomes (KEGG) [39]. There were 71.4 and 33.9% genes assigned to KEGG Orthology (KO) database and KEGG pathways, respectively. Principal coordinate analysis (PCoA) of all KO genes showed that the fiber-rich and starch-rich diets selected for different metabolic functions (Fig. 3A), with “carbohydrate metabolism pathways” being the most abundant category that was significantly different between diets (Additional Fig. S4).

We screened for carbohydrate-active enzymes (CAZymes) in the assembled metagenomic contigs (Additional File S2). High starch intake increased the abundance of total CAZymes (*p* = 0.01), including auxiliary activities (AAs), carbohydrate-binding module (CBMs), and glycosyltransferases (GTs) (Fig. 3B). Furthermore, the rank of the top five carbohydrate-active enzyme classes and top ten assigned phyla or genera differed between the two treatments (Additional Fig. S5). The GHs had the highest relative abundance among the six CAZyme classes, and the abundance of GH subfamilies was significantly different between the two dietary treatments (Fig. 3C). The fiber-rich diet selected for a greater abundance of *β*-xylosidase GH43 (*p* = 0.001, 1.3-fold higher), which was assigned to *Prevotella*, *Bacteroides*, and *Fibrobacter* at the genus level (Additional Fig. S6). Abundance of *α*-amylase GH13 was greater with the starch-rich diet (Fig. 3D, Additional File S3, *p* < 0.001, 1.4-fold higher) and was assigned to *Prevotella*, *Ruminobacter*, and *Clostridium* (Additional Fig. S6). Thus, both dietary treatments promoted bacterial communities with distinct capacities to digest carbohydrates.

We then analyzed the abundance of genes encoding for enzymes related to the pathways of acetate, propionate, butyrate, and methanogenesis (Additional File S4). The fiber-rich diet enriched for the pathways of acetate production (*pta* gene), acetate to butyrate production (*acs* gene), propionate production via succinate as intermediate (*MUT* and *sdhA* genes), and methanogenesis (*mcrA* and *mcrB* genes), while the starch-rich diet enriched for the acrylate pathway of propionate production (*ldh* and *pct* genes) (Fig. 3E, Additional Fig. S7, *p* < 0.05). *Bacteroidota*, *Firmicutes*, and *Proteobacteria* were the major phyla assigned to the acrylate pathway in the starch-rich diet. With the fiber-rich diet, *Fibrobacterota* was prominent in fermentative acetate and propionate production, and as expected, *Firmicutes* was the major phylum for acetate to butyrate production pathway, and *Methanobacteriota* (*Euryarchaeota*) was the assigned phylum for methanogenesis (Fig. 3E, Additional File S5).

### Distinct hydrogen production and incorporation pathways are selected by two diets

**Fig. 4:**
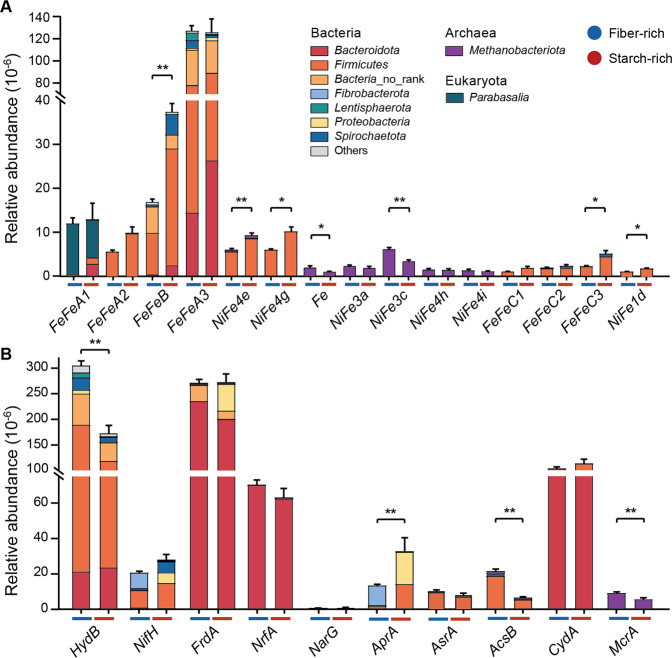
Fiber-rich and starch-rich treatment resulted in different hydrogenase and terminal reductases level in rumen microbiome. **A** Hydrogenase genes distributions assigned by phylum; **B** genes of associated terminal reductases distributions assigned by phylum. Fermentative hydrogenases (group B, A1 and A2 FeFe-hydrogenases), electron-bifurcating hydrogenases (group A3 and A4 FeFe-hydrogenases), energy-converting hydrogenases (bidirectional; group 4a, 4c, 4d, 4e, 4f and 4g NiFe-hydrogenases), methanogenic hydrogenases (Fe-hydrogenases, group 3a, 3c, 4h, 4i and 1k NiFe-hydrogenases), respiratory hydrogenases (group 1a, 1b, 1c, 1d, 1k, 2a), sensory hydrogenases (group C FeFe-hydrogenases). HydB hydrogenase-associated diaphorase. NifH nitrogenase. H_2_ uptake pathways can be coupled to fumarate reduction (FrdA fumarate reductase), nitrate ammonification (NrfA, ammonia-forming nitrite reductase; NarG, dissimilatory nitrate reductase; NapA, periplasmic nitrate reductase), sulfate and sulfite reduction (AprA adenylylsulfate reductase; AsrA alternative sulfite reductase; DsrA, dissimilatory sulfite reductase), dimethyl sulfoxide and trimethylamine N-oxide reduction (DmsA DMSO and TMAO reductase), reductive acetogenesis (AcsB, acetyl-CoA synthase), aerobic respiration (CydA cytochrome *bd* oxidase), and methanogenesis (McrA methyl-CoM reductase). No rank means there is no specific taxonomic information at the phylum level. Only genes of average relative abundance > 1 were shown, while other were shown in additional file S6. Significance was tested using independent two-group Wilcoxon rank-sum tests. Data with error bars are expressed as mean ± standard error. **p < 0.01, ***p < 0.001, *n* = 12/group.

Given the significant differences observed in VFA profiles, dH_2_ concentrations, and CH_4_ production (Fig. 1B, C), we screened for the genes encoding for the catalytic subunits of H_2_-producing and H_2_-consuming enzymes in the assembled contigs. Of the 2,686 genes identified, 82%, 17% and 1.2% were annotated as [FeFe]-, [NiFe]- and [Fe]-hydrogenases respectively. Hydrogenases were taxonomically assigned to 149 genera, including *Bacteroides*, *Clostridium*, *Oscillibacter*, *Methanobrevibacter*, and *Ruminococcus* (Additional File S6). As the two diets exhibited distinct hydrogenase composition (Additional Fig. S8), we then classified hydrogenases into subgroups. The trimeric group A3 [FeFe]-hydrogenases, which mediate the process of electron confurcation during fermentative carbohydrate degradation leading to H_2_ production [40], were the most abundant hydrogenases with both treatments (Fig. 4A). The diaphorase subunit (HydB) of these hydrogenases was highly enriched with the fiber-rich diet (*p* < 0.01, 1.8-fold higher), and was primarily encoded by *Firmicutes* and *Bacteroidota* at the phylum level (Additional File S6).

The fiber-rich diet enriched genes for methanogenic hydrogenases (group 3a [NiFe]-hydrogenases and [Fe]-hydrogenases) encoded by *Methanobacteriota* (Fig. 4A, *p* < 0.05, 1.5-fold higher). The starch-rich diet enriched genes encoding for fermentative group B [FeFe]-hydrogenases (*p* < 0.01, 2.2-fold higher), energy-converting group 4e and 4g [NiFe]-hydrogenases, respiratory group 1d [NiFe]-hydrogenases, and sensory group C3 [FeFe]-hydrogenases (Fig. 4A, *p* < 0.05), which were taxonomically assigned mainly to *Firmicutes* and *Spirochaetota* (Additional File S6). These results suggest the starch-rich diet resulted in enhanced H_2_ flow, which was consistent with greater fermentation and higher rumen dH_2_ concentration (Fig. 1B).

We further analyzed signature genes that support hydrogenotrophic growth, including methanogenesis, acetogenesis, fumarate reduction, sulfidogenesis, nitrate reduction, and aerobic respiration [5]. The fiber-rich diet selected for signature genes associated with methanogenesis, including methyl-CoM reductase (*mcrA*, Fig. 4B, *p* = 0.009, 1.6-fole higher), which were mainly affiliated with *Methanobrevibacter* (Additional File S6). The starch-rich diet selected for a higher abundance of group 1d [NiFe]-hydrogenases (*p* < 0.05) encoded by *Firmicutes*, a membrane-bound type of enzymes known to support hydrogenotrophic respiration, along with the signature gene for sulfate reduction (*aprA*, *p* = 0.002, 3.0-fold higher). An unexpected result was that the marker gene for hydrogenotrophic acetogenesis (acetyl-CoA synthase, *acsB*, *p* < 0.01) was threefold more abundant with the fiber-rich diet (Fig. 4B). Acetyl-CoA synthase is a reversible enzyme, for example mediating acetate utilization in various sulfate-reducing bacteria [41], but most reads (about 75%) were most closely related to those of acetogenic *Firmicutes*.

### Genome mapping and in vitro fermentation verifies microbial functions

The Hungate1000 collection of assembled genomes, which includes virtually all of the bacterial and archaeal species that have been cultivated from the rumen of diverse ruminant species [30, 42], was used to gain a stronger strain-level understanding of the findings of this study. One million random reads from each sample were extracted and aligned to the Hungate1000 genomes, resulting in an average mapping rate of 20.2% (Additional Fig. S9). We found that 257 and 168 genomes were more enriched by the fiber-rich and starch-rich diets, respectively (Additional Fig. S10). Genomes encoding for GH43 were more enriched with the fiber-rich diet and assigned to *Bacteroides*, *Prevotella*, and *Fibrobacter*, while genomes encoding for GH13 were enriched with the starch-rich diet and assigned to *Ruminobacter* and *Clostridium*. The fiber-rich diet selected 8 out of 10 putatively methanogenic genomes, which harbor unique hydrogenases (group 3a, 3c, 4h, 4i [NiFe]-hydrogenases and [Fe]-hydrogenases) and the signature gene *mcrA*, as well as putatively 5 out of 8 hydrogenotrophic acetogenic bacterial genomes encoding electron-bifurcating hydrogenases (group A3, A4 [FeFe]-hydrogenases) or the signature gene *acsB*. The starch-rich diet selected for 67 out of 130 genomes encoding fermentative hydrogenases (group A1, A2, and B [FeFe]-hydrogenases), assigned to *Lachnospirales* and *Selenomonadales*, and 17 out of 33 genomes encoding for respiratory hydrogenases (group 1 and 2a [NiFe]-hydrogenases) assigned to *Selenomonadales*. These genomes with terminal sulfate and sulfite reductases were more enriched with the starch-rich diet, and mainly assigned to *Lachnospirales* (Additional File S7).

**Fig. 5:**
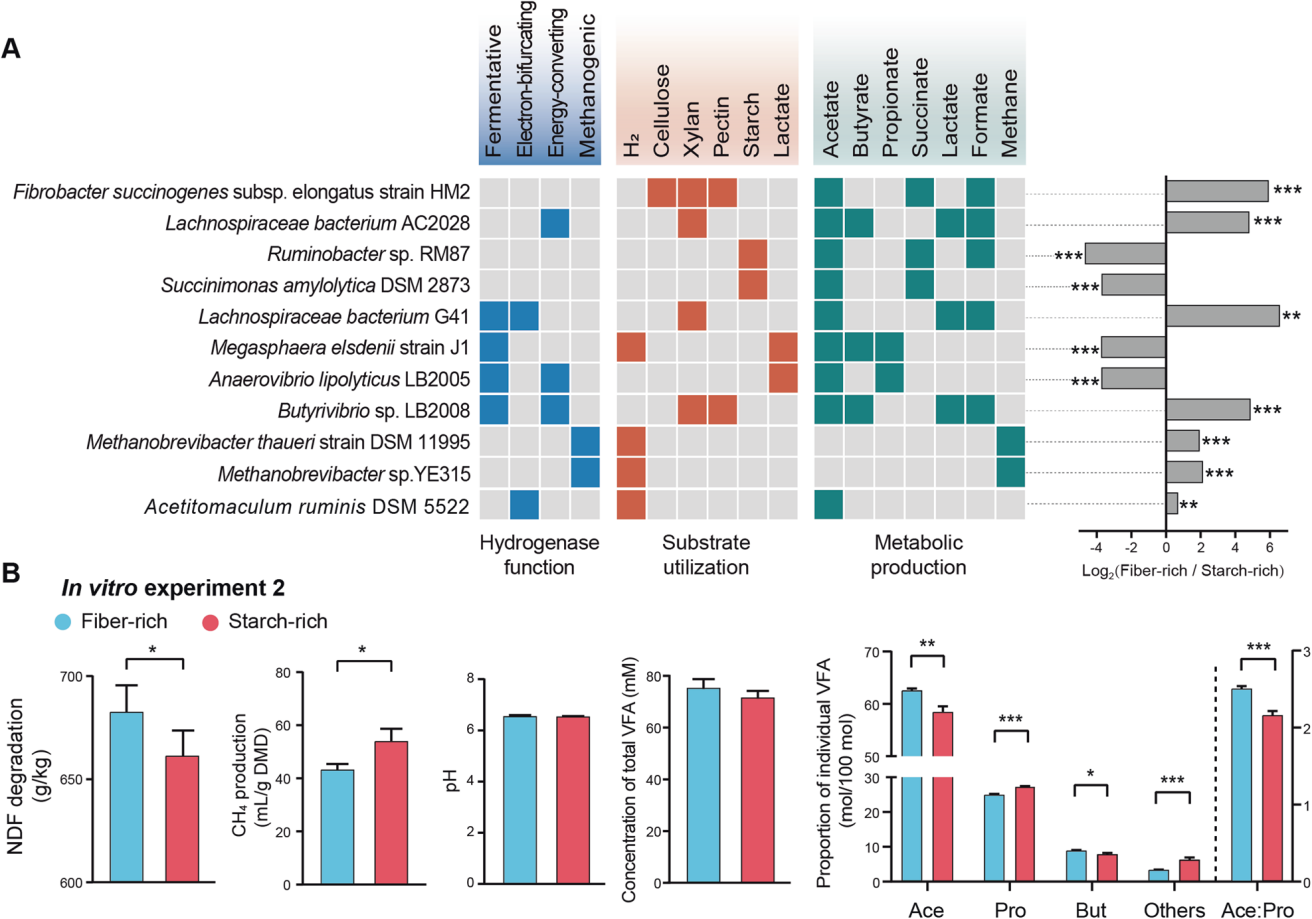
Metabolic features enriched in microbiome of fiber-rich and starch-rich diets in relation to function. **A** metabolic features of representative microorganisms. Reads from each sample were aligned to sequenced genomes of cultured rumen microorganisms in the Hungate1000 collection using the burrows-wheeler alignment tool. The hydrogenase function, substrate utilization, and metabolite production of each microorganism based on the known growth characteristics [5, 30] are colored in blue, orange, and green, respectively. The ratios between alignments of fiber-rich/starch-rich samples to each genome are presented, and data were expressed as log2 (ratio). Genomes attributes: fibrolytic bacteria, *Fibrobacter succinogenes* subsp. *elongatus* strain HM2 and *Lachnospiraceae bacterium* AC2028; amylolytic bacteria, *Ruminobacter* sp. RM87 and *Succinimonas amylolytica* DSM 2873; acetate producer, *Lachnospiraceae bacterium* G41 and *Fibrobacter succinogenes* subsp *elongatus* strain HM2; butyrate producer, *Lachnospiraceae bacterium* AC2028 and *Butyrivibrio* sp. LB2008; lactate utilizer and propionate producer, *Megasphaera elsdenii* strain J1 and *Anaerovibrio lipolyticus* LB2005; hydrogenotrophic methanogen, *Methanobrevibacter thaueri* strains DSM 11995 and sp. YE315; acetogens, *Acetitomaculum ruminis* DSM 5522. Detailed information of other genomes is shown in additional file S7; **B** methane (CH_4_) production, neutral detergent fiber (NDF) degradation, pH and volatile fatty acid (VFA) profile of in vitro rumen experiment 2 with rice straw fermentation by inoculating fiber-rich or starch-rich selected microbiome. Data with error bars are expressed as mean ± standard error. **p < 0.01, ***p < 0.001, *n* = 12/group.

We performed an analysis of ranks of attributes based on differential abundance between two treatments. *Fibrobacter succinogenes* subsp. *elongatus* strain HM2 and *Lachnospiraceae bacterium* AC2028, both of which have functions in fiber degradation [30], were the most representative genomes enriched with the fiber-rich diet (*p* < 0.001). Both *Ruminobacter* sp. RM87 and *Succinimonas amylolytica* DSM 2873, known to degrade starch [30], were the most representative genomes with the starch-rich diet (Fig. 5A, *p* < 0.001, Additional Fig. S11). We validated by q-PCR that 16S rRNA gene copies of *F. succinogenes* and *Ruminobacter amylophilus* were the more abundant in the fiber-rich and starch-rich treatments, respectively (Additional Fig. S12, *p* < 0.001). Enhanced fiber degradation was further verified by in vitro experiment 2, which indicated that inoculating microbiome of fiber-rich diet resulted in greater NDF degradation (Fig. 5B, *p* = 0.026).

We selected the two most differential abundant genomes of microorganisms inferred to produce acetate, propionate, or butyrate (Additional File S7). The microbiome of the fiber-rich diet favored acetate and butyrate production while being enriched with *Lachnospiraceae bacterium* G41 and AC2028, *F. succinogenes* subsp. *elongatus* strain HM2, and *Butyrivibrio* sp. LB2008 (*p* < 0.01), while the microbiome of the starch-rich diet favored propionate production with lactate as an intermediate along with enrichment of *Megasphaera elsdenii* strain J1 and *Anaerovibrio lipolyticus* LB2005 (*p* < 0.001, Additional Fig. S11). These results were further verified by in vitro experiment 2 by incubating rice straw inoculated with fiber-rich or starch-rich selected microbiomes (Fig. 5B). Accordingly, five of six representative genomes encoded fermentative, electron-bifurcating and energy-converting hydrogenases, while one genome (i.e., *Megasphaera elsdenii* strain J1) had the capacity to use H_2_ as a substrate for VFA production (Fig. 5A). These results further upheld that dietary carbohydrate selected for microbiomes with distinct pathways of VFA production and H_2_ metabolism.

We selected genomes encoding marker genes for hydrogenotrophic methanogenesis (*mcrA*) and acetogenesis (*acsB*). The most differentially abundant genomes were *Methanobrevibacter thaueri* strains DSM 11995 and sp. YE315, as well as the hydrogenotrophic acetogen *Acetitomaculum ruminis* DSM 5522 [43], which were all enriched with the fiber-rich diet (Fig. 5A and Fig. S11, *p* < 0.01, Additional File S7). Both *M. thaueri* strains DSM 11995 and sp. YE315 encode methanogenic hydrogenases to use H_2_ to reduce CO_2_ to CH_4_, while *A. ruminis* DSM 5522 encodes electron-bifurcating group A3 [FeFe]-hydrogenases predicted to use H_2_ to reduce CO_2_ to acetate (Fig. 5A). The *acsB* gene is also encoded in some butyrate-producing bacteria (*e.g*., *Eubacterium limosum*), which produces acetate through the Wood–Ljungdahl pathway and converts it to butyrate [44, 45]. In turn, in vitro experiment 2 where rice straw was the sole substrate indicated that inoculation of the fiber-rich selected microbiome resulted in lower CH_4_ and greater acetate and butyrate production compared to the starch-rich selected microbiome (Fig. 5B, *p* < 0.05). This suggests the possibility that the fiber-rich diet may have selected for hydrogenotrophic acetogens, which may use some of the H_2_ that would otherwise be used for methanogenesis to produce acetate.

## Discussion

**Fig. 6:**
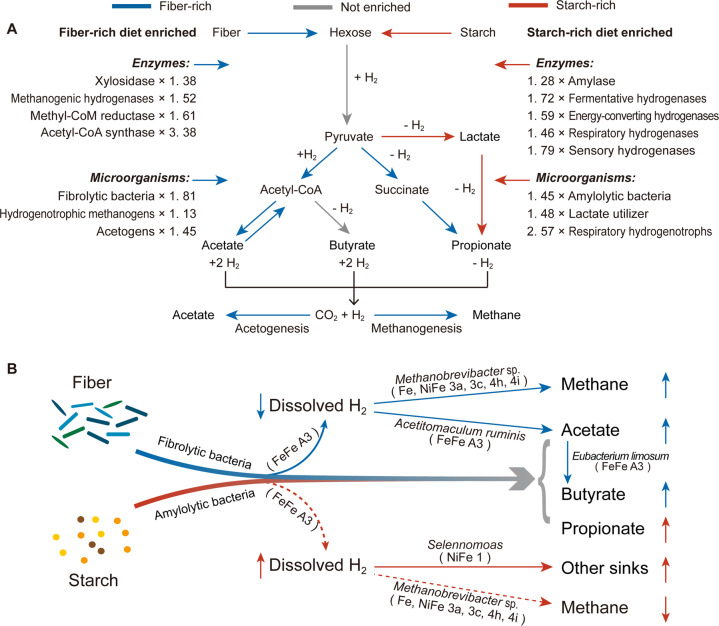
Fiber-rich or starch-rich diet exhibits distinct microbiome carbohydrate and hydrogen metabolic pathways in bovine rumen. **A** consolidating analysis on enzymes, microorganisms of Hungate1000 collection, metabolites and pathways recruitment. “+ H_2_”, H_2_ produced; “- H_2_”, H_2_ consumed. The fold changes (left: fiber-rich/starch-rich; right: starch-rich/fiber-rich) of enzymes or microorganisms in each group are labeled besides, and presented in additional file S8 in details; **B** proposed rumen H_2_ metabolism models. Representative species or genus and corresponding hydrogenases are shown next to the arrow, and extracted from Figs. 1B, C, 4A, 5, and additional file S8. The solid and dotted line (square) indicated trends of enhanced and reduced pathways, respectively. H_2_ hydrogen. The upward arrows, increased metabolites; the downward arrows, decreased metabolites.

The fiber-rich diet selected for fibrolytic bacteria (e.g., *Fibrobacter* and *Ruminococcus*) and enriched for genes encoding for xylosidases (e.g., GH43, Fig. 6A). The characteristic microbiome and metabolic strategies promoted by the fiber-rich diet are likely to be important for ruminants living in harsh environmental conditions, allowing them to digest and ferment low-quality plant biomass to produce VFA to be used by the host animal for maintenance, reproduction, and growth. Tibetan ruminants, which are well adapted to digest poor-quality forage diet [15, 46], have been shown to harbor more fibrolytic bacteria than lowland ruminants, especially of *Ruminococcus albus* (100-fold higher) and *F. succinogenes* (50-fold higher) [47]. The camel, living in the barren desert steppe with access to the lignified coarsest parts of scarce plants, has likewise greater enrichment of fiber-degrading rumen bacteria compared to domestic ruminants such as cattle and sheep [48, 49]. Furthermore, fibrolytic bacteria can also be enriched in the hindgut of non-ruminants including pigs [50], horses [51], and rabbits [52] when the fiber content of the diet is increased. Proliferation of fibrolytic bacteria for enhanced fiber utilization can be an important metabolic strategy for host animals to adapt to eating lignified diets. However, enrichment in fibrolytic bacteria with the fiber-rich treatment could be associated with increased CH_4_ production, leading to a reduction in dietary energy use efficiency.

The first important finding of this work is that a fiber-rich diet increased the abundance of the *acsB* gene, encoding for acetyl-CoA synthase of hydrogenotrophic acetogens (e.g., *Acetitomaculum* spp.), compared to a starch-rich diet. Hydrogenotrophic acetogens have been isolated from the rumen and foregut microbial ecosystems of cattle and kangaroos fed with high forage diets and from the hindgut of wood-eating termites [14, 43, 53]. Some ruminal hydrogenotrophic acetogens (e.g., *Acetitomaculum*) have the ability to simultaneously use inorganic (H_2_/CO_2_) and organic substrates (e.g., cellobiose and glucose) as energy and carbon sources, whereas others (*e.g*., *Blautia* sp. Ser8) do not use H_2_ in the presence of glucose, producing acetate as the sole reduced end product [16, 54]. One discrepancy with other studies is that we observed greater abundance of *Spirochaetota* in the starch-rich diet, whereas *Spirochaetota* have been shown to be stimulated by fiber-rich diets and can be hydrogenotrophic acetogens in other animal hosts [55–57]. It is unclear if our findings are compounded by the specificity of the primers used to profile community composition. Nevertheless, the metagenomic and activity-based analyses suggest an increase in the abundance of acetogenic bacteria, in addition to fermentative acetate production, can also contribute to increased acetate production with the fiber-rich diet. As some butyrate-producing bacteria (e.g., *Eubacterium limosum*) encode the *acsB* gene for acetyl-CoA synthase, a greater proportion of butyrate produced by fiber-rich selected microbiome can go through the acetate pool.

In turn, this work provides novel insights about the possible role of hydrogenotrophic acetogenesis in the rumen microbial communities selected by the fiber-rich diet. Studies report that methanogens can outcompete for acetogens due to their higher affinity for H_2_ and lower Gibbs energy change of H_2_ utilization for methanogenesis compared to hydrogenotrophic acetogenesis [7, 58]. The classical theory holds that stimulation of acetogens may be an effective strategy for redirection of metabolic hydrogen towards acetate production in methanogenesis-inhibited scenarios with increased ruminal H_2_ concentration [5]. However, we found an increased abundance of the marker gene for hydrogenotrophic acetogenesis (e.g., *acsB*) with lower dH_2_ concentration in the fiber-rich diet. Our in vitro experiment 2 with rice straw as the sole substrate indicated that inoculation with fiber-rich selected microbiome paradoxically promoted acetate production with decreased CH_4_ production in comparison to the starch-rich selected microbiome. These results suggest that a greater community of hydrogenotrophic acetogens in the fiber-rich selected microbiome might perhaps compete for H_2_ with methanogens. There has been speculation that high-altitude ruminants yak and Tibetan sheep might favor acetate production with lower CH_4_ formation compared to lowland cattle and sheep [15, 59]. Molecular hydrogen incorporation into hydrogenotrophic acetogenesis is estimated to contribute for about 1% of acetate formed and account for 1 to 2% of total reducing equivalents incorporated in in vitro cultures inoculated with sheep rumen fluid [60]. The stimulation of acetate synthesized by acetogens would not only decrease the emissions of greenhouse gas CH_4_, but would also help improving energy use efficiency for host animals. Such pathways of energy harvesting may be a competitive strategy for ruminants fed poor-quality diets.

High starch intake generally results in decreased pH, and even subclinical or lactic rumen acidosis [17]. We found that gradually increasing dietary starch up to 90% did not drop the pH below 6.0 or harm the rumen epithelium, although lactate concentration significantly increased. The enrichment of *M. elsdenii* and *A. lipolyticus*, which use the acrylate pathway to convert lactate to propionate, is likely important for avoiding acidosis. *M. elsdenii* reportedly utilizes 60–80% of the lactate produced in the rumen [61], and accounts for 20% of lactate utilizers in the rumen of animals fed high-concentrate diets [62]. *A. lipolyticus* can also utilize lactate and produce propionate as an end product [63, 64]. In addition to preventing lactate accumulation [17, 65], lactate conversion to propionate incorporates reductant into NADH, thus competing with H_2_ formation and ultimately methanogenesis [66]. A strong community of bacteria metabolizing lactate to propionate via acrylate can prevent lactate accumulation and help maintaining a healthy rumen.

A second important finding of this work is that, in both fiber-rich and starch-rich selected microbiomes, electron-bifurcating group A3 [FeFe]-hydrogenases were inferred to be the primary mediators of ruminal H_2_ production. This result coincides with the findings about the importance of H_2_ formation by electron confurcation in the rumen [67]. Equally important is the differential abundance of hydrogenases and its relationship with methanogenesis promoted by the two diets. The fiber-rich diet resulted in low rumen dH_2_ concentration and increased abundance of the HydB subunit of electron-bifurcating group A3 [FeFe]-hydrogenases. The starch-rich diet caused higher rumen dH_2_ concentration and increased the abundance of genes encoding fermentative group B [FeFe]-hydrogenases, as well as energy-converting 4e and 4g [NiFe]-hydrogenases, which couple ferredoxin oxidation to H_2_ production in anaerobic bacteria, including in *Firmicutes* and *Spirochaetota* (Fig. 6A). Elevated rumen dH_2_ concentration with the starch-rich diet was accompanied with decreased abundance of genes encoding methanogenic [NiFe] and [Fe]-hydrogenases, as well as methyl-CoM reductases, which mediate hydrogenotrophic methanogenesis, which is in line with decreased CH_4_ production. A mechanism explaining less CH_4_ formation from concentrates based on faster rate of growth and/or lower maximal growth rate of methanogens resulting in greater H_2_ concentration, and consequently acetate and H_2_ formation becoming thermodynamically less favorable, with eventually less H_2_ being available for CH_4_ formation, has been proposed before [7]. Our finding of decreased abundance of methanogenic hydrogenases with the starch-rich diet is in line with that proposal, as fewer methanogens and methanogenic enzymes would presumably be present in the rumen with the faster rumen outflow rates and lower rumen pH associated to feeding concentrates. Greater H_2_ concentrations in turn thermodynamically favors H_2_-incorporating pathways such a propionate production, which agrees with the increased abundance of respiratory 1d [NiFe]-hydrogenases and putative sensory C3 [FeFe]-hydrogenases affiliated to *Firmicutes*, and supports hydrogenotrophic respiration (e.g., *Selenomonas*) [5].

Overall, our results indicate that the type of carbohydrate fed to ruminants alters rumen microbiome composition and function with distinct H_2_ metabolism, which is closely associated with rumen methanogenesis, and VFA production and profile (Fig. 6B). The fiber-rich diet enriched for fibrolytic bacteria and enhanced fiber utilization, and acetate and H_2_ production, along with increased methanogens abundance and greater CH_4_ production and decreased H_2_ concentrations. Enhanced acetate production by the fiber-rich selected microbiome might partly result from hydrogenotrophic acetogenesis, and, if confirmed, would be a novel metabolic strategy for ruminants to use H_2_ and CO_2_ with greater efficiency for the host compared to CH_4_ production. The starch-rich diet enriched for amylolytic bacteria, and enhanced propionate production through the acrylate pathway with lactate as an intermediate, helping to maintain a healthy rumen and decrease production of H_2_ available for methanogenesis. High starch intake increased fermentation with decreased methanogens abundance, and caused an increase in rumen dH_2_ concentration accompanied by greater abundance of hydrogenases involved in hydrogenotrophic respiration. These findings provide novel insights of distinct H_2_ metabolic pathways in rumen microbiota of cattle to adapt to interventions based on feeding carbohydrates with contrasting fermentability, and help to understand energy harvesting strategies, healthy rumen maintenance, and CH_4_ mitigation in ruminants. More work is needed to elucidate the adaptation, resilience, and colonization of bacteria with the metabolic flexibility to use H_2_ in the rumen, and their benefit for host animals.

## Supplementary information

Supplementary materials

Dataset 1

Dataset 2

Dataset 3

Dataset 4

Dataset 5

Dataset 6

Dataset 7

Dataset 8

## Data Availability

All data are available in the main text or the in supplementary materials. Raw reads of 16S rRNA gene sequencing of ruminal microbiota are available at National Center for Biotechnology Information (NCBI, project number PRJNA736529). Raw reads of metagenomic sequencing of ruminal microbiota are available at NCBI (project number PRJNA779163).
