# Supplementary material for: Dietary selection of metabolically distinct microorganisms drives hydrogen metabolism in ruminants
Source: ISME J. 2022 Aug 5;16(11):2535–46. doi: 10.1038/s41396-022-01294-9 (PMC9562222; doi:10.1038/s41396-022-01294-9)
Supplement: Supplementary file 1 — Supplementary materials [file 41396_2022_1294_MOESM1_ESM.docx]

**Supplementary information**

**Supplementary Files (Excel files)**

**File S1:** The Mean Decrease Accuracy at genus level.

**File S2:** Fiber-rich and starch-rich treatments alter the relative abundance of CAZyme families enriched in rumen microbiome.

**File S3:** Fiber-rich and starch-rich treatments alter the relative abundance of GH families enriched in rumen microbiome.

**File S4:** EC and KO numbers that analyzed in this study.

**File S5:** Fiber-rich and starch-rich treatments alter phylogenetic distribution of sequences of the KEGG pathways enriched in rumen microbiome.

**File S6:** Fiber-rich and starch-rich treatments alter phylogenetic distribution of sequences of hydrogenase and terminal reductase genes enriched in rumen microbiome.

**File S7:** Profiles of the Hungate1000 genomes.

**File S8:** Consolidation of results from enzymes and genomes (Hungate1000 collection) recruitment analysis.

**Supplementary Figures**


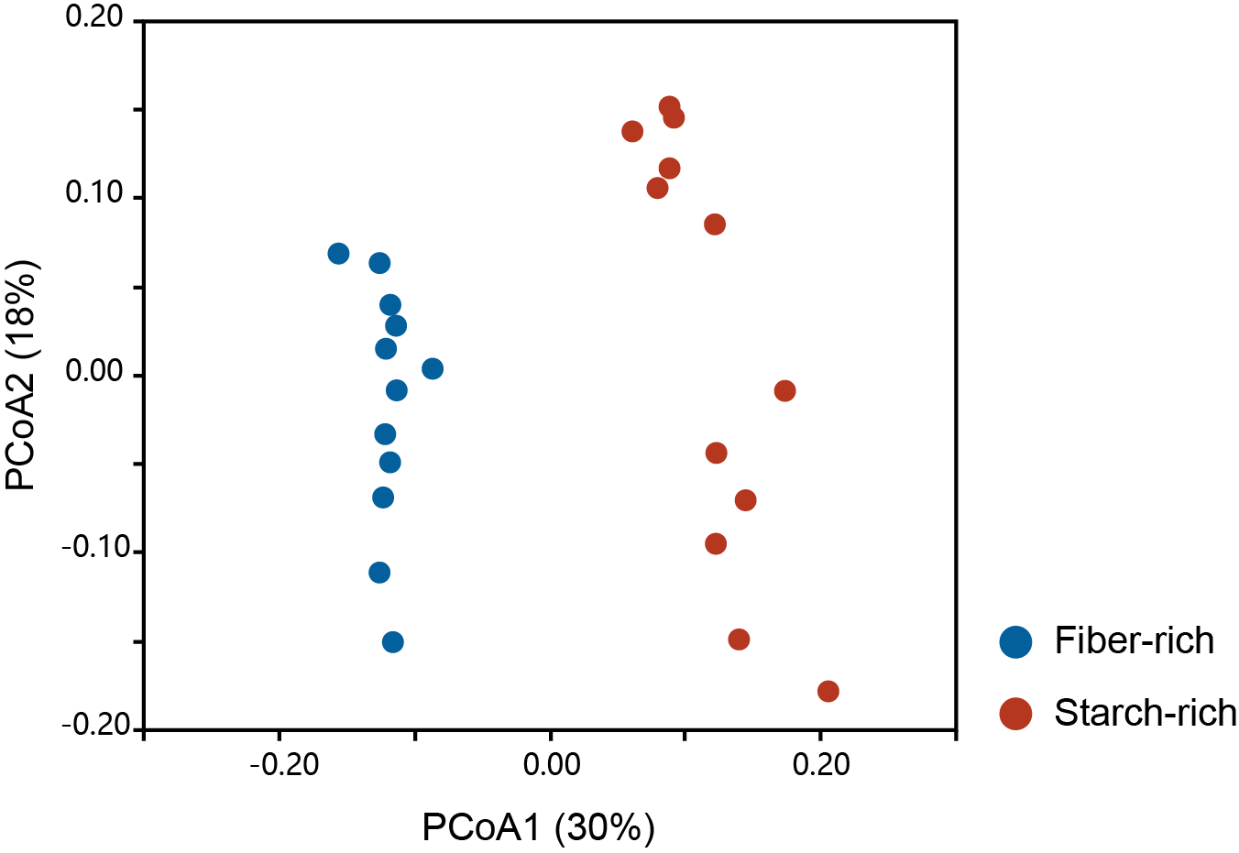


**Figure S1.** Principal coordinate analysis profile of ruminal bacterial community based on Bray-Curtis dissimilarity matrix at genus level referred to Kraken-minidb database. PERMANOVA, *p* = 0.001, *R*^2^ = 0.73, *n* = 12/group.


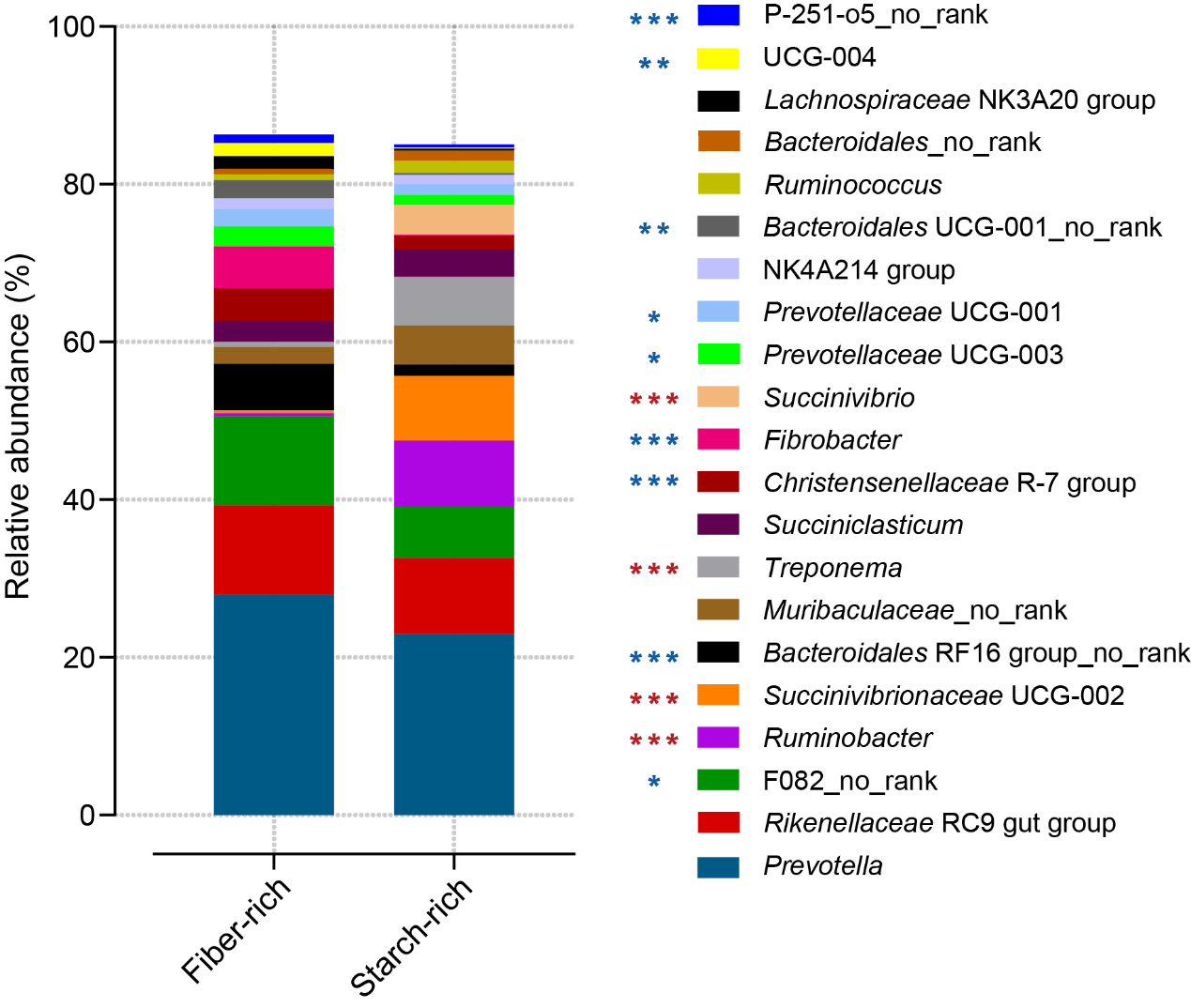


**Figure S2.** Comparison of relative abundance of the genera in rumen bacterial community of fiber-rich or starch-rich diet. Only genera with the average relative abundance more than 1% in at least one group were analyzed. Norank means there is no specific taxonomic information at the genus level. **p* < 0.05, ***p* < 0.01, ****p* < 0.001, *n* = 12/group.


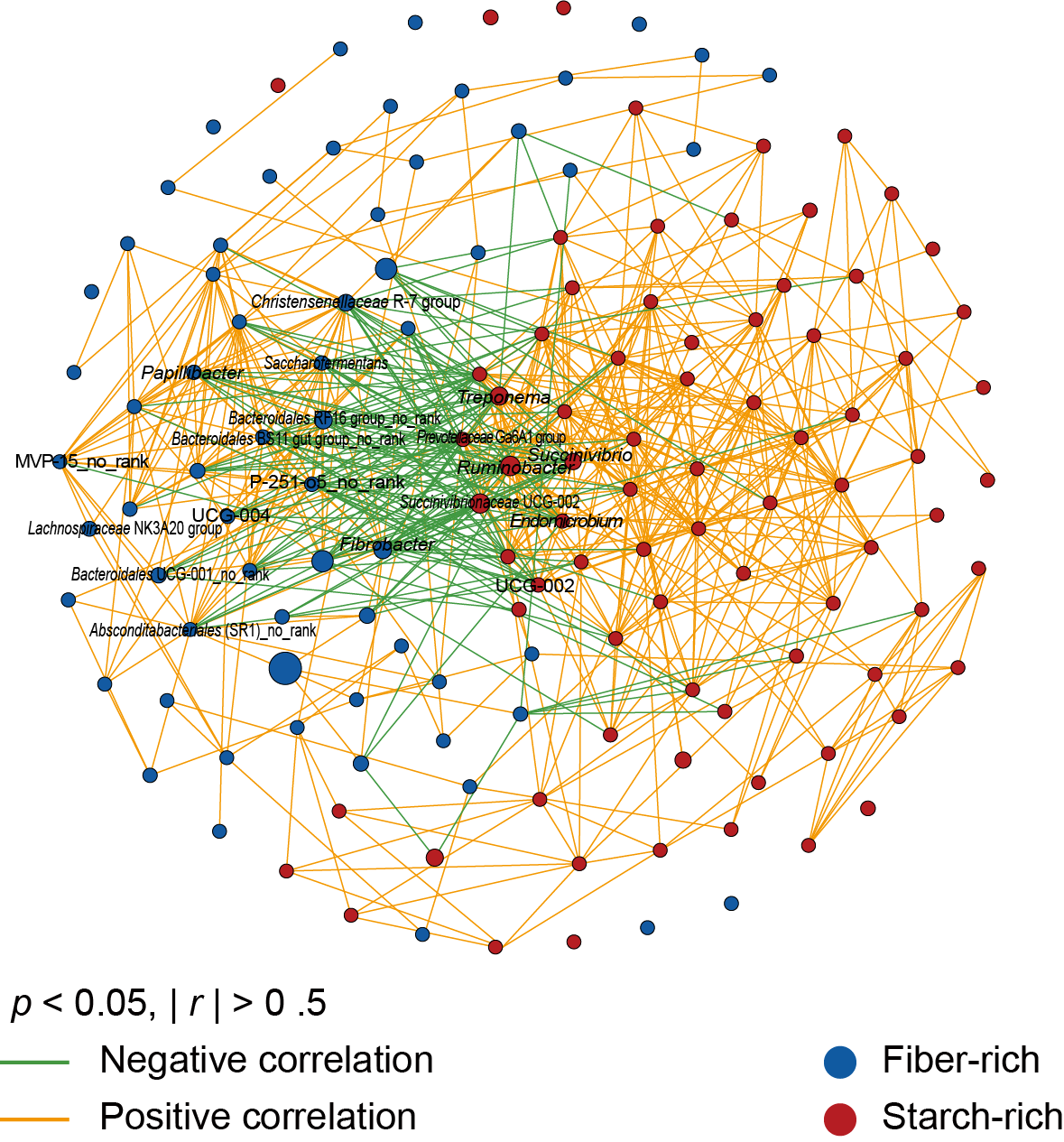


**Figure S3.** The Spearman’s correlation network of bacteria at the genus level in rumen bacterial community of fiber-rich or starch-rich diet. The size of nodes indicates the relative abundance. Taxa of representative 20 bacterial genera are specified based on the rank of their relative importance. A line denotes correlation between two genera (*p* < 0.05, Spearman’s | *r* | > 0.5). No_rank means there is no specific taxonomic information at the genus level. *n* = 12/group.


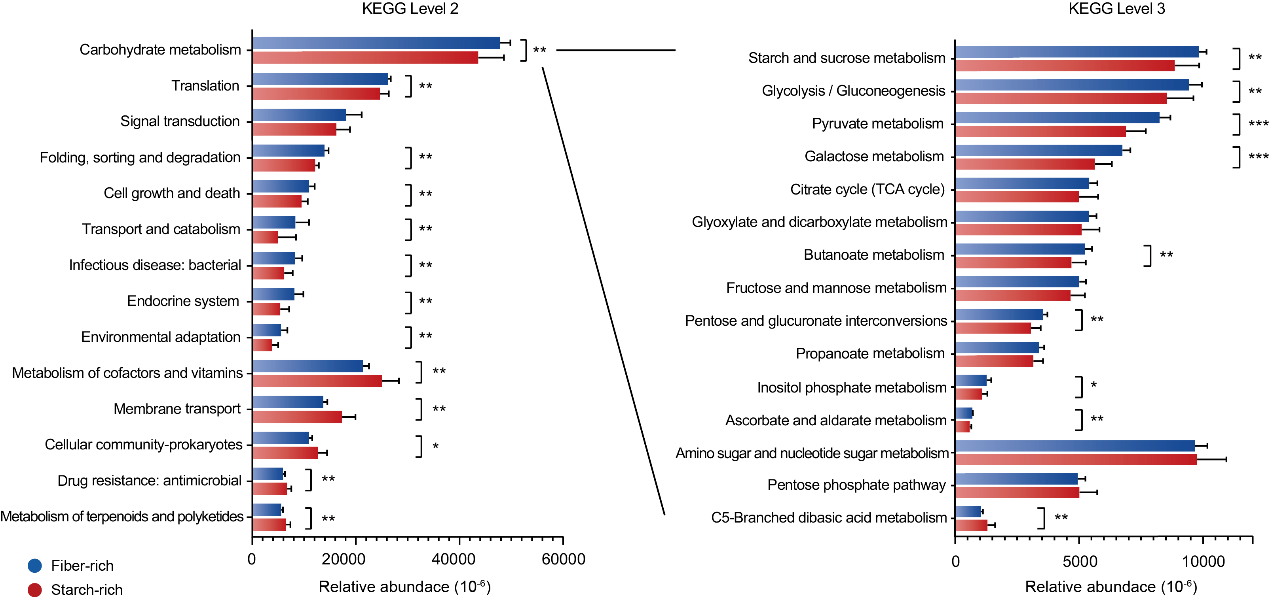


**Figure S4.** Comparison of genes of KEGG level 2 and 3 pathways enriched in rumen microbiomes of fiber-rich or starch-rich diet. Data with error bars are expressed as mean ± standard error. **p* < 0.05, ***p* < 0.01, ****p* < 0.001, *n* = 12/group.


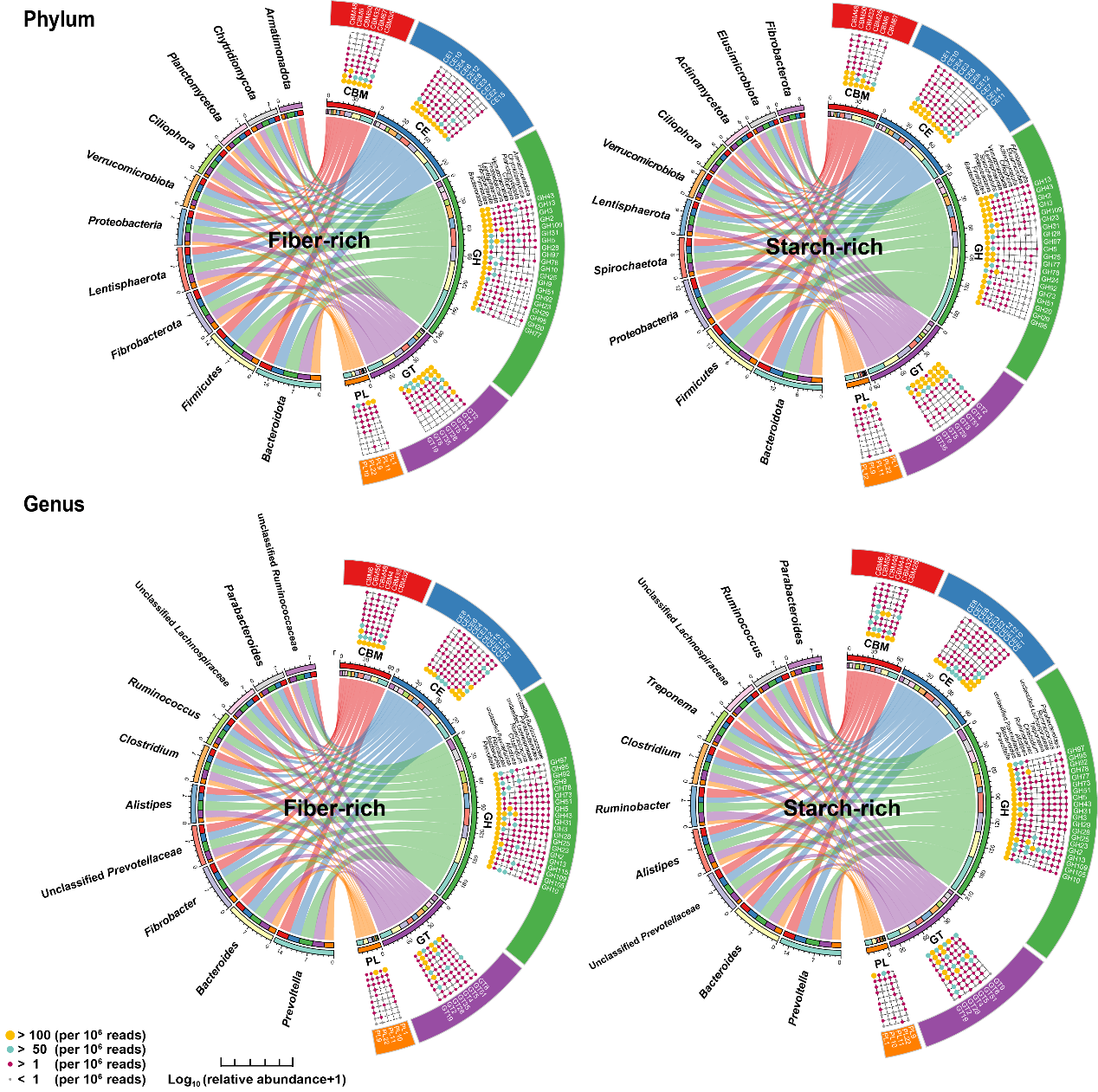


**Figure S5.** Phylogenetic distribution of top-5 GH family enzymes assigned to the identified top-10 identified phyla or genera in rumen microbiomes of fiber-rich or starch-rich diet. CBM, carbohydrate binding module; CE, carbohydrate esterase; GH, glycoside hydrolase; GT, glycosyltransferase; PL, polysaccharide lyase. Unclassified means that the classification information for the sequences was not found in the database. *n* = 12/group.


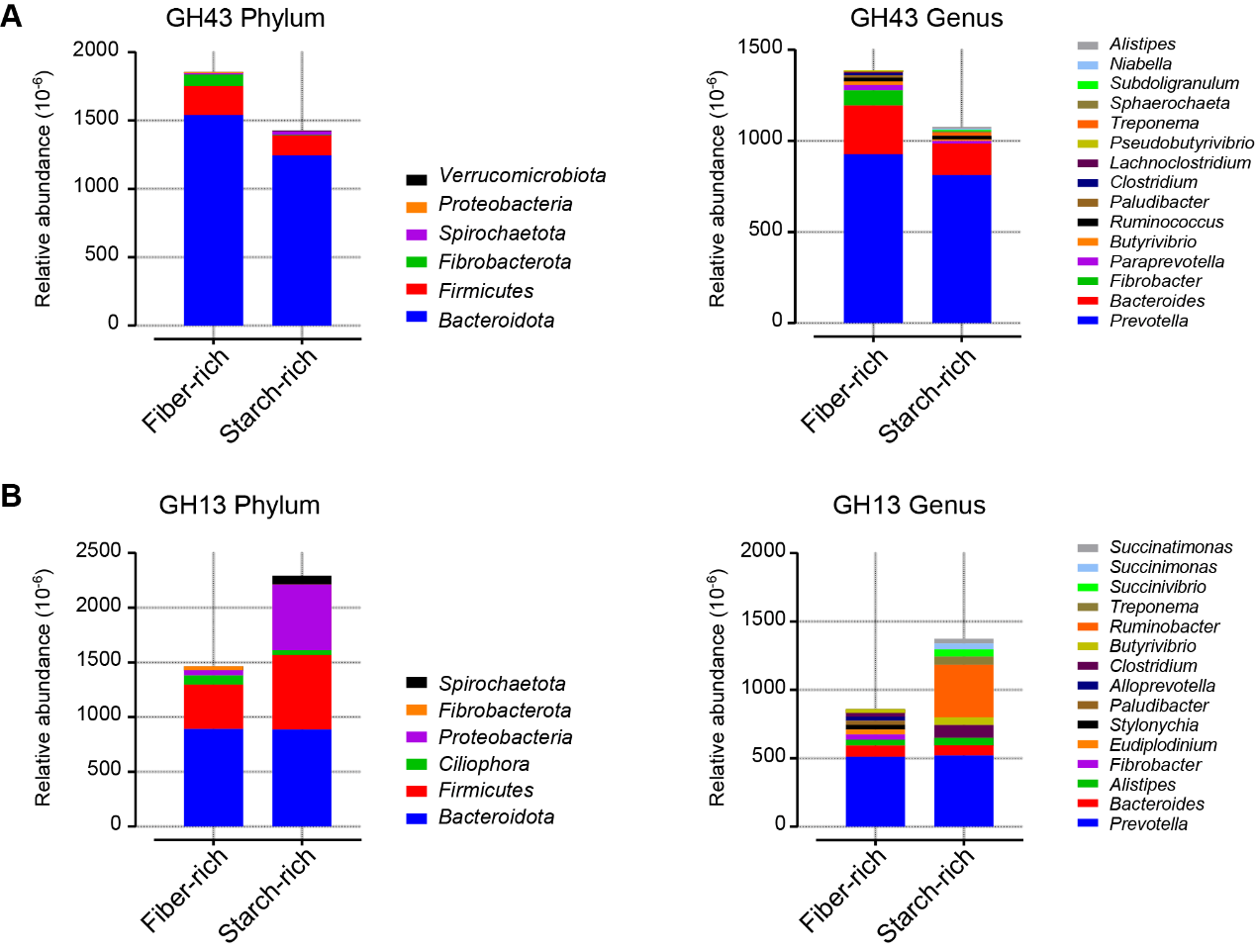


**Figure S6.** Phylogenetic distribution of GH43 (A) and GH13 (B) assigned to the identified phylum and genus in rumen microbiome of fiber-rich or starch-rich diet. *n* = 12/group.


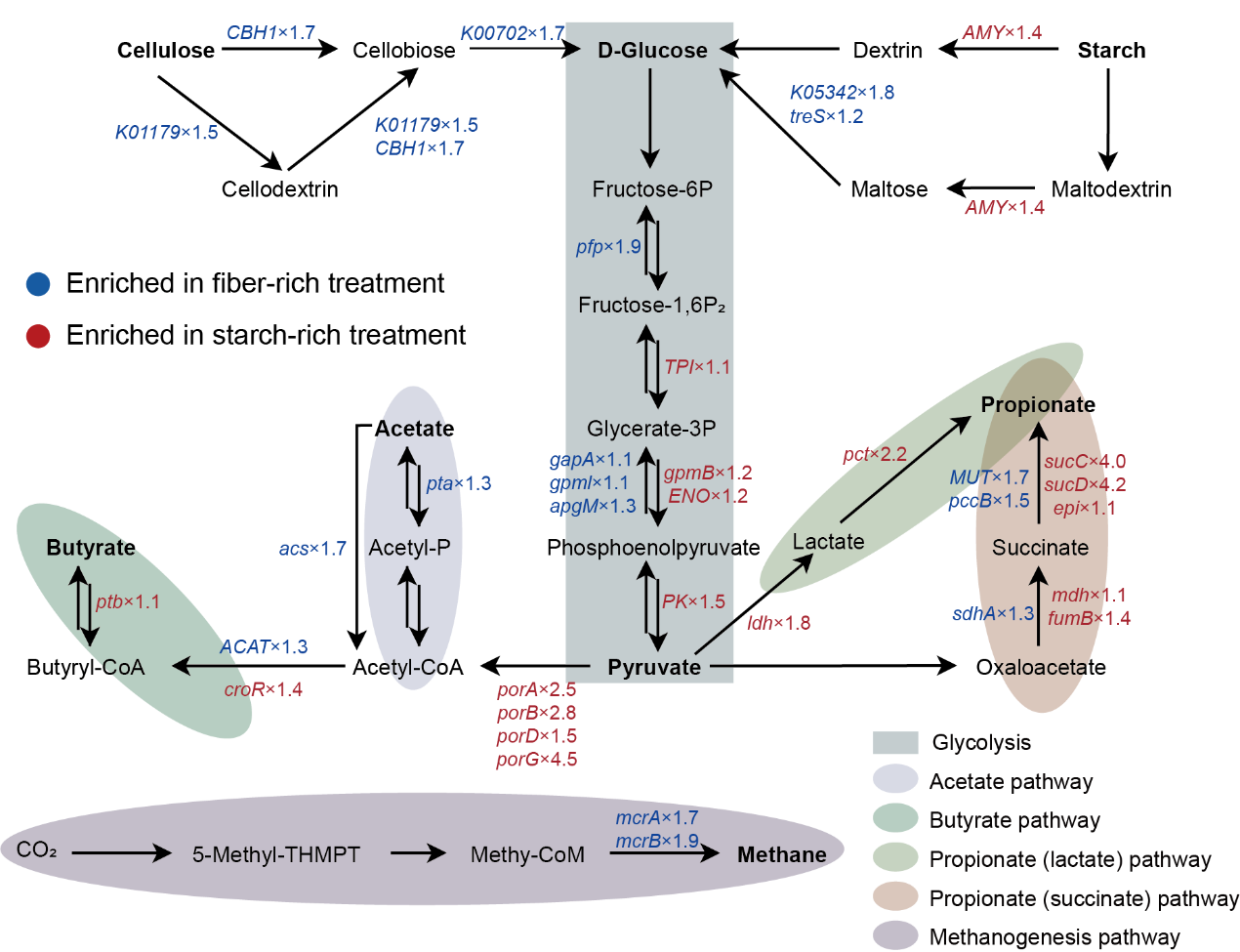


**Figure S7.** Comparisons by Wilcoxon test of the relative abundance of KO enzymes enriched in rumen microbiome regarding carbohydrates metabolism to produce volatile fatty acids and methane between the fiber-rich and starch-rich dietary treatments. Fold changes (blue: fiber-rich / starch-rich; red: starch-rich / fiber-rich) of enzymes in each group are labeled besides each reaction arrow. *n* = 12/group.


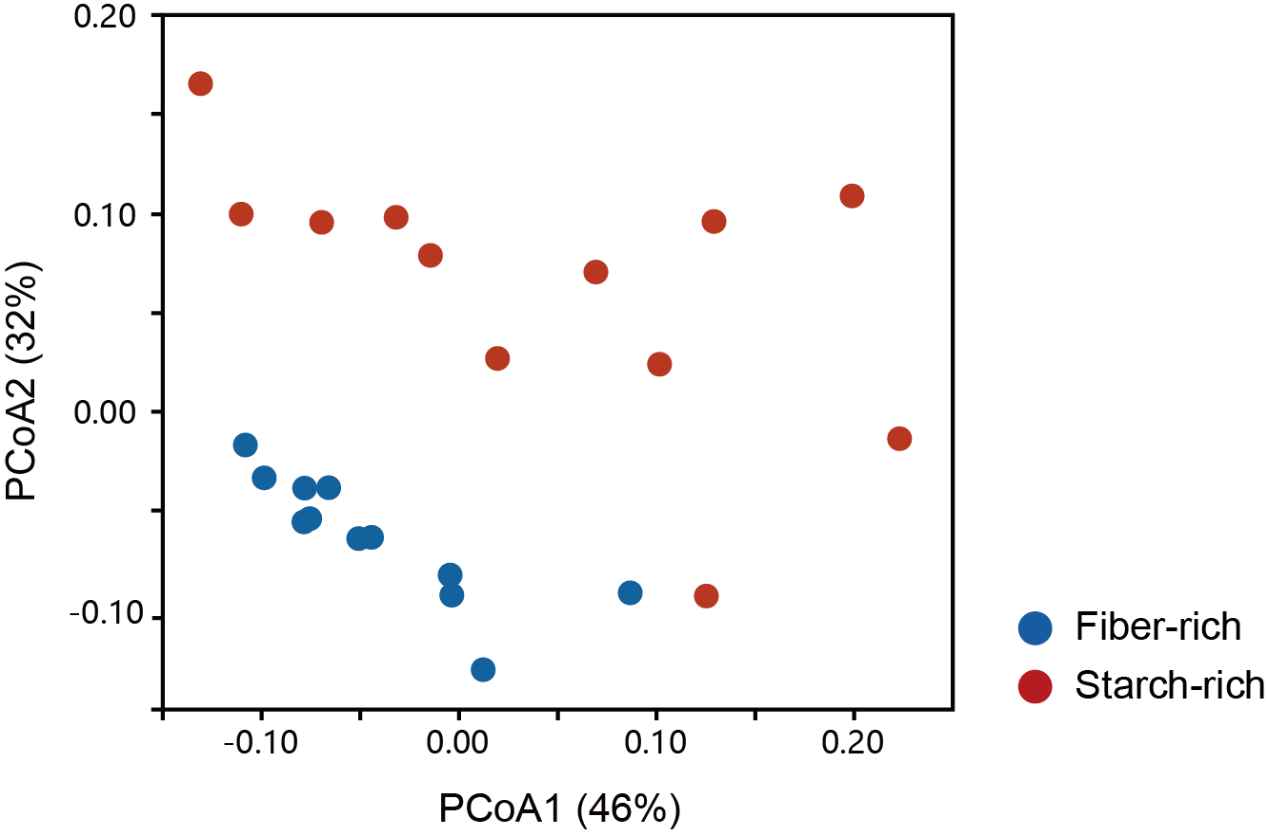


**Figure S8.** Principal coordinate analysis profile of hydrogenases abundance based on Bray-Curtis dissimilarity matrix. PERMANOVA, *p* <0.001, *R*^2^=0.29, *n* = 12/group.

**
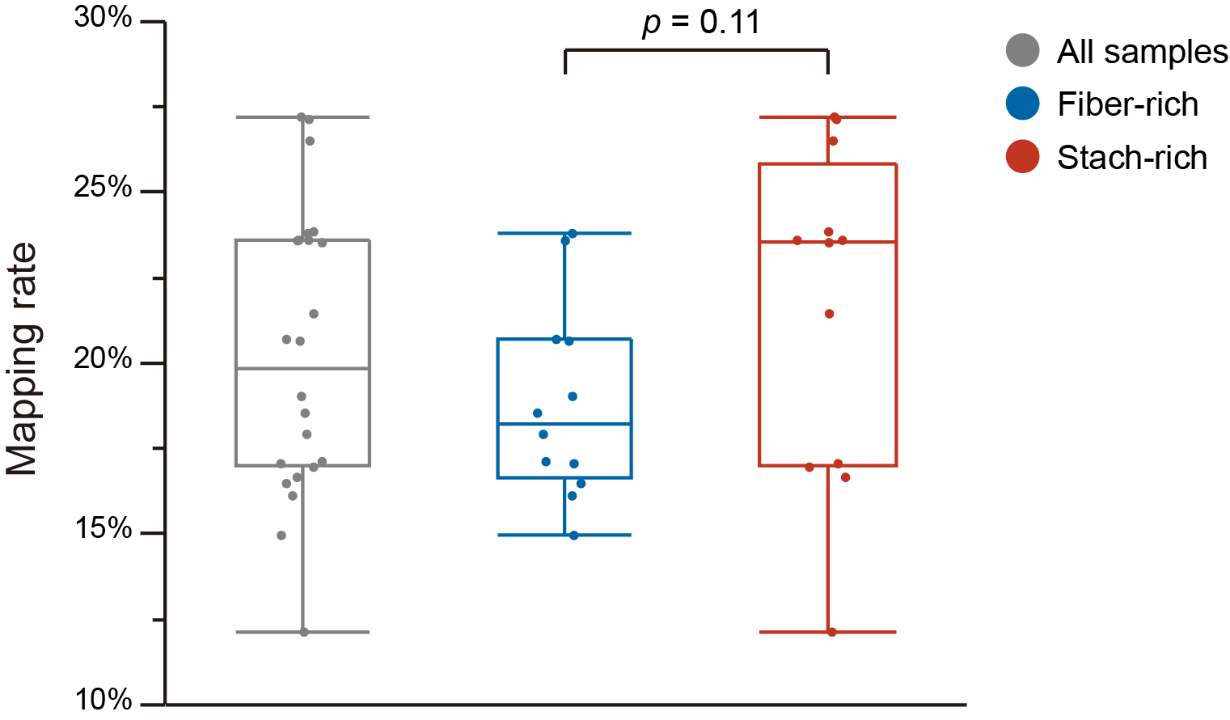
**

**Figure S9.** Mapping rate of the 24 metagenomes to Hungate1000 genomes.


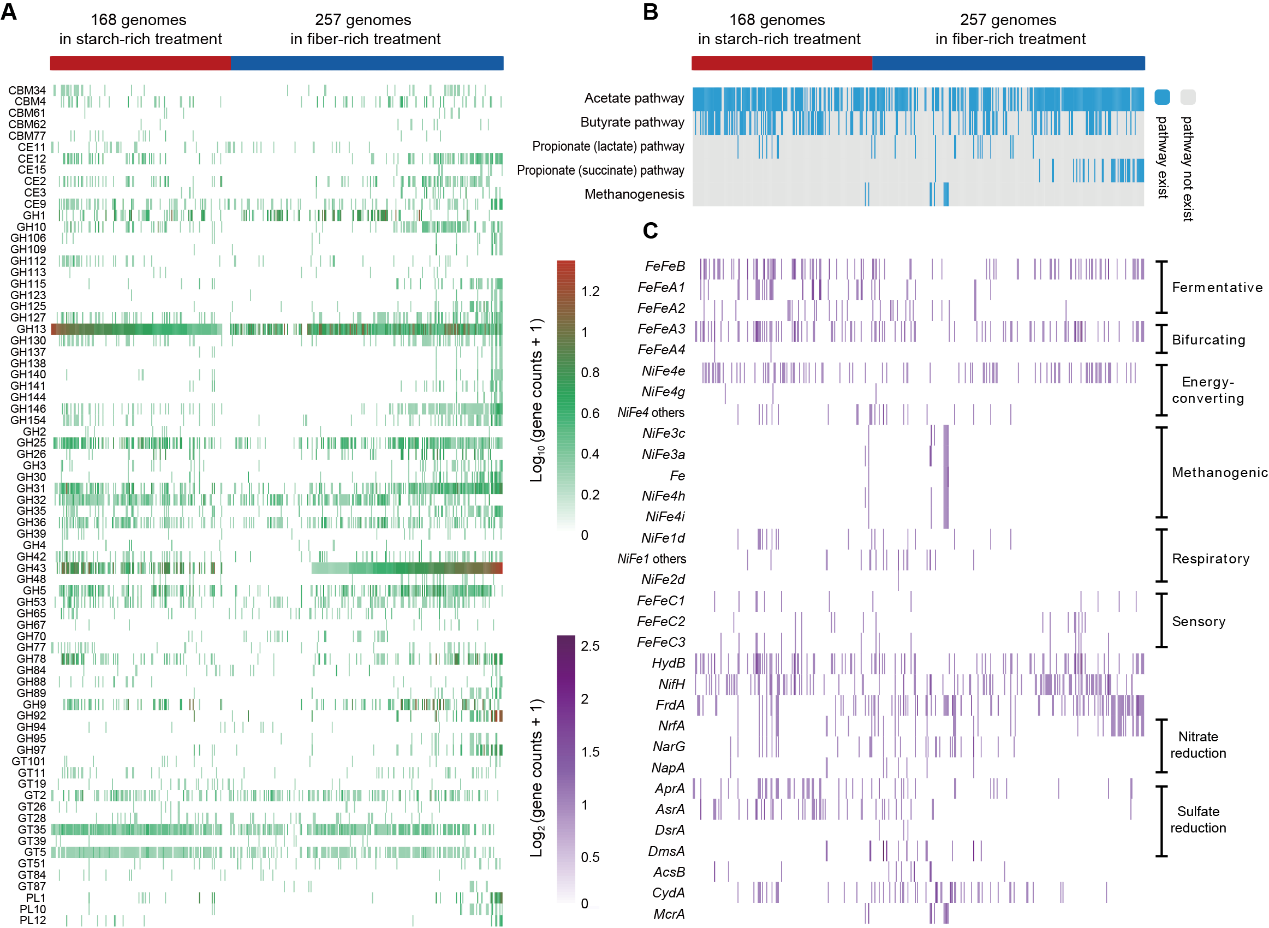


**Figure S10.** Heatmaps for selected gene of CAZymes (A), KEGG pathways (B), hydrogenases and associated terminal reductases (C) of identified 425 differentially enriched genomes in rumen microbiome of fiber-rich or starch-rich diet. Reads from each sample were aligned to sequenced genomes of cultured rumen microorganisms of Hungate1000 database using the burrows-wheeler alignment tool, and the most prevalent assigned genera in these genomes were denoted on the top. CAZyme genes with more than 10 genomes encoded were shown. *n* = 12/group.


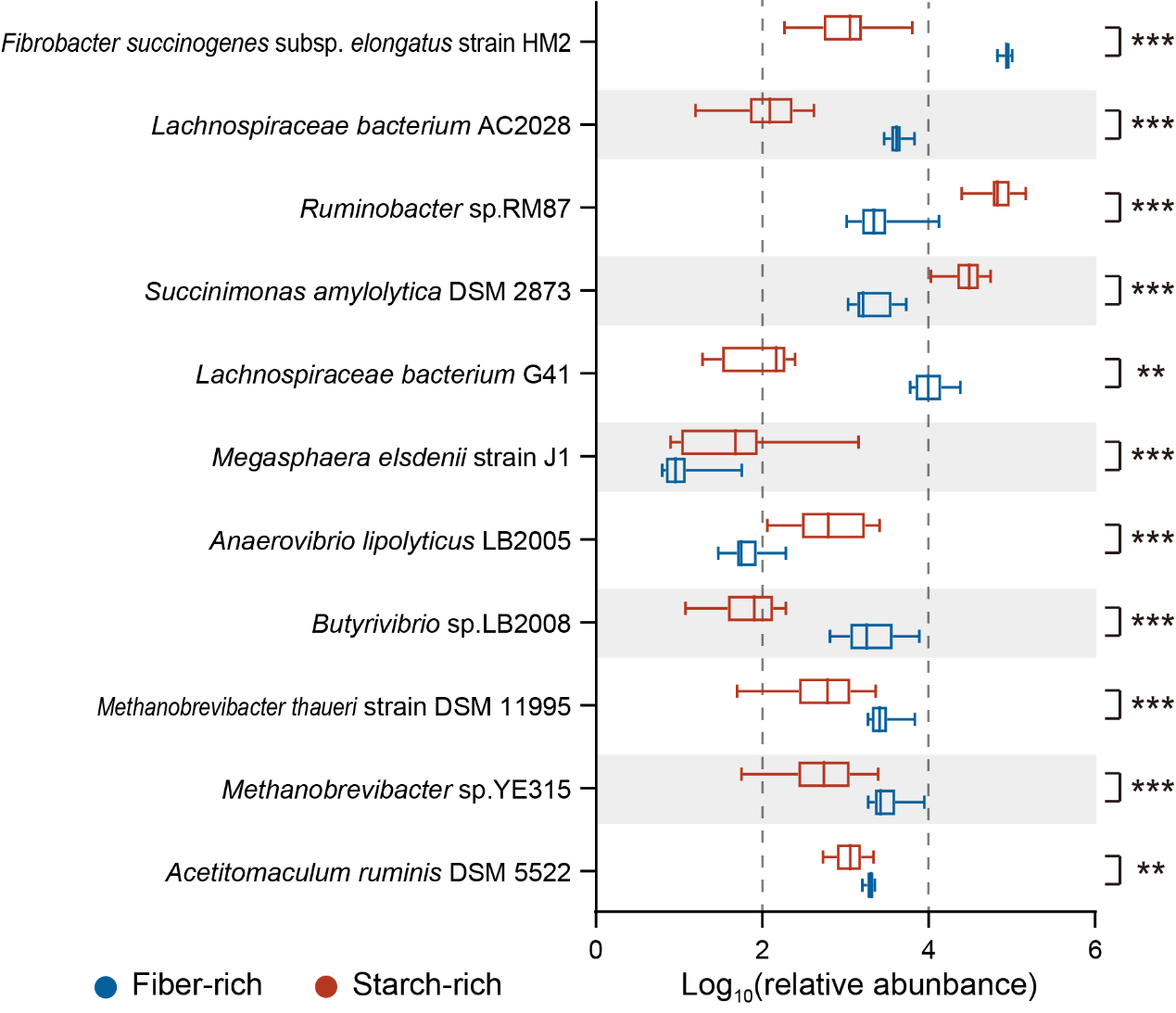


**Figure S11.** Comparison of relative abundance of the representative genomes between the fiber-rich and starch-rich diets. ***p* < 0.01, *** *p* < 0.001, n = 12/group.


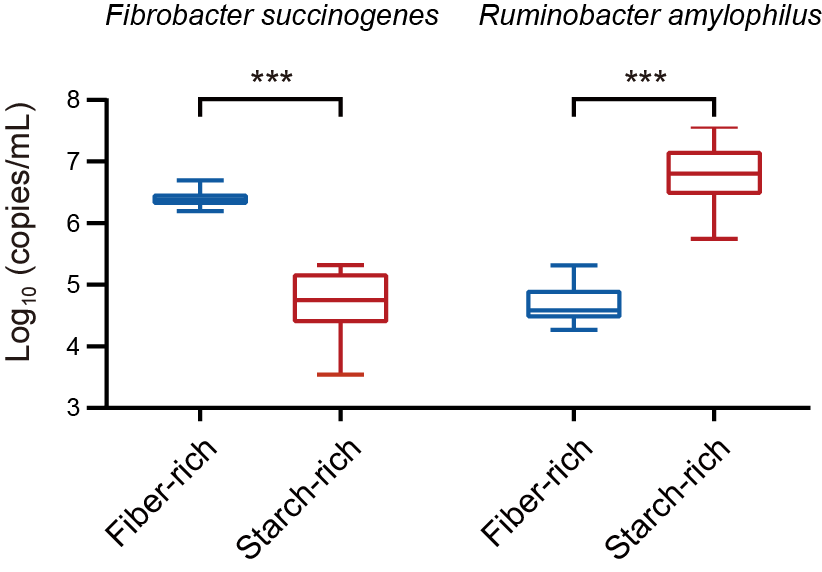


**Figure S12.** 16S rRNA copy numbers of *Fibrobacter succingenes* and *Ruminobacter amylophilus* in rumen microbiomes of animals fed the fiber-rich or starch-rich diet. *** *p* < 0.001, n = 12/group.

**Supplementary Tables**

**Table S1. Ingredients and chemical compositions of fiber-rich or** **starch-rich diets.**

| Item | Fiber-rich diet | Starch-rich diet ^1^ | | |
| --- | --- | --- | --- | --- |
|  |  | Period 1 | Period 2 | Period 3 |
| Ingredient composition (g/kg DM) | | | | |
| Forage content | 700 | 500 | 300 | 100 |
| Corn stover silage ^2^ | 600 | 400 | 200 | 0 |
| Rice straw ^3^ | 100 | 100 | 100 | 100 |
| Concentrate | 300 | 500 | 700 | 900 |
| Soyabean meal | 140 | 92 | 102 | 122 |
| Maize meal | 45 | 291 | 481 | 665 |
| Wheat bran | 30 | 30 | 30 | 30 |
| Puffing urea | 10 | 12 | 12 | 8 |
| Soybean oil | 20 | 20 | 20 | 20 |
| Premix ^4^ | 55 | 55 | 55 | 55 |
| Nutrient composition, g/kg DM | | | | |
| OM | 920 | 924 | 928 | 933 |
| Ash | 80 | 76 | 72 | 67 |
| NDF | 476 | 383 | 311 | 234 |
| Forage NDF | 416 | 343 | 291 | 234 |
| ADF | 215 | 291 | 100 | 117 |
| CP | 142 | 141 | 140 | 139 |
| Starch^2^ | 96 | 264 | 393 | 517 |
| Starch/NDF | 0.20 | 0.69 | 1.26 | 2.29 |

ADF, acid detergent fiber; CP, crude protein; DM, dry matter; NDF, neutral detergent fiber; OM, organic matter;

^1^ Starch-rich diets were formulated by gradually replacing 1/3 of corn stover silage with maize meal, and starch-rich diets of period 1, 2 and 3 contains 50%, 70% and 90% concentrate respectively. Each period was last for 100 d.

^2^ Corn stover silage contained 438 g/kg of DM, 60 g/kg of CP, 606 g/kg of NDF, 265 g/kg ADF and 72 g/kg starch on DM basis

^3^ Rice straw contained 967 g/kg of DM, 40 g/kg of CP, 661 g/kg of NDF, 396 g/kg of ADF and 70 g/kg of starch on DM basis.

^4^ The premix (vitamins and microelements) was formulated to provide (per kg of DM): 5 g of NaCl, 10 g of yeast culture, 1 000 000 IU of vitamin A, 100 000 IU of vitamin D, 682 IU vitamin E, 4364 mg of Zn, 44 mg of Se, 65 mg of I, 1091 mg of Fe, 22 mg of Co, 1364 mg of Mn, and 1091 mg of Cu.

† Mean values from the analysis of three samples.

**Table S****2. Primer sequences of differentially expressed genes related to VFA transporters and intracellular pH regulation in the rumen epithelium of bovine for q-PCR.**

| Gene Name | Gene ID | Primer sequence (5′→3′) | Amplicon Size (bp) |
| --- | --- | --- | --- |
| *HMGCL* | NM_001075132 | Forward: TCCACGAGACGGAsssCTACAAAA  Reverse: AGAGGCGGCTCCAAAGATG | 277 |
| *HMGCS*-1 | AY581197.1 | Forward: AGAGGATCGGCGTGTTTTCTT  Reverse: CAGACCCTGGTGTGGCATCT | 87 |
| *HMGCS*-2 | BC112666.1 | Forward: TCTGGCCCATCACTCTGCC  Reverse: AGTGGGGAGCCTGGAGAAGC | 126 |
| *NHE*-1 | NM_174833 | Forward: CCTCTACAGCTACATGGCCTAC  Reverse: GGGAGATGTTGGCTTCCA | 113 |
| *NHE*-2 | XM_604493 | Forward: TTGGAGAGTCCCTGCTGAAC  Reverse: GGCCGTGATGTAGGACAAAT | 257 |
| *NHE*-3 | AJ131764 | Forward: AGCTACGTGGCCGAGGG  Reverse: AGACAGAGGCCTCCACGGT | 121 |
| *MCT*-1 | NM_001037319 | Forward: ACCAGTTTTAGGTCGTCTCA  Reverse: GGCTTCTCAGCAACATCTACA | 207 |
| *β-actin* | NM_173979 | Forward: CTAGGCACCAGGGCGTAATG  Reverse: CCACACGGAGCTCGTTGTAG | 177 |
| *GAPDH* | NM_001034034 | Forward: TGGAAAGGCCATCACCATCT  Reverse: CCCACTTGATGTTGGCAG | 60 |

*HMGCL*, 3-Hydroxy-3-methylglutaryl-CoA lyase; *HMGCS-*1, 3-Hydroxy-3-methylglutaryl-CoA synthase, isoform 1; *HMGCS-*2, 3-Hydroxy-3-methylglutaryl-CoA synthase, isoform 2; *NHE*-1, Na^+^/H^+^ exchanger 1; *NHE*-2, Na^+^/H^+^ exchanger 2; *NHE*-3, Na^+^/H^+^ exchanger 3 *MCT*-1, Monocarboxylate transporter, isoform 1; *GAPDH*: glyceraldehyde-3-phosphate dehydrogenase.

**Table S****3. Primer sequences for q-PCR of target species.**

| Target specie | Primer sequence (5′→3′) | Amplicon Size (bp) |
| --- | --- | --- |
| *Fibrobacter succingenes* | Forward: GTTCGGAATTACTGGGCGTAAA  Reverse: CGCCTGCCCCTGAACTATC | 121 |
| *Ruminobacter amylophilus* | Forward: CAATAAGCATTCCGCCTGGG  Reverse: TTCACTCAATGTCAAGCCCTGG | 102 |

**Table S****4. Comparisons of fermentation end products in the rumen samples collected at three periods between fiber-rich and starch-rich treatments (**n = 12/group).

| Item ^1^ | 0h | | 2.5h | | 6h | | SEM  SEM | *P* | | |
| --- | --- | --- | --- | --- | --- | --- | --- | --- | --- | --- |
|  | Fiber-rich | Starch-rich | Fiber-rich | Starch-rich | Fiber-rich | Starch-rich |  | Diet | Time | Diet×Time |
| **First period** | | | | | | | | | | |
| pH | 7.10 | 7.01 | 6.76 | 6.70 | 7.48 | 7.11 | 0.060 | 0.06 | < 0.001 | 0.24 |
| Headspace gas |  |  |  |  |  |  |  |  |  |  |
| Hydrogen, μM | 19.1 | 20.5 | 29.6 | 45.2 | 26.1 | 25.7 | 3.61 | 0.28 | 0.02 | 0.40 |
| Methane, M | 10.29 | 9.88 | 9.01 | 9.25 | 8.58 | 9.92 | 0.308 | 0.37 | 0.09 | 0.22 |
| Dissolved hydrogen, μM | 0.58 | 0.43 | 3.77 | 6.21 | 2.40 | 6.57 | 0.969 | 0.02 | < 0.001 | 0.047 |
| Lactate, mM^1^ | 0.58 | 0.57 | 0 | 0 | 0 | 0 | 0.007 | 0.819 | < 0.001 | 0.819 |
| VFA concentration, mM | 80.4 | 82.1 | 117.7 | 127.0 | 101.9 | 106.5 | 3.74 | 0.05 | < 0.001 | 0.55 |
| Molar percentage of individual VFA, mol/100 mol | | | | | | | | | | |
| Acetate | 61.1 | 60.0 | 56.3 | 56.7 | 58.5 | 58.9 | 0.36 | 0.78 | < 0.001 | 0.18 |
| Propionate | 18.4 | 17.9 | 21.8 | 20.9 | 20.9 | 18.5 | 0.47 | 0.05 | < 0.001 | 0.69 |
| Butyrate | 15.2 | 16.2 | 16.3 | 16.6 | 15.9 | 17.2 | 0.30 | 0.07 | 0.10 | 0.71 |
| Others | 5.3 | 5.9 | 5.6 | 5.8 | 4.7 | 5.4 | 0.14 | 0.003 | 0.001 | 0.30 |
| Acetate to propionate ratio | 3.33 | 3.41 | 2.60 | 2.75 | 2.81 | 3.23 | 0.08 | 0.07 | < 0.001 | 0.72 |
| **Second period** | | | | | | | | | | |
| pH | 7.20 | 6.90 | 7.17 | 6.89 | 7.22 | 6.96 | 0.037 | < 0.001 | 0.36 | 0.88 |
| Headspace gas |  |  |  |  |  |  |  |  |  |  |
| Hydrogen, μM | 33.9 | 36.0 | 54.3 | 71.8 | 31.0 | 50.2 | 5.78 | 0.13 | 0.03 | 0.52 |
| Methane, M | 13.7 | 12.0 | 15.2 | 13.4 | 14.5 | 12.9 | 0.30 | 0.01 | 0.03 | 0.97 |
| Dissolved hydrogen, μM | 0.55 | 3.32 | 4.24 | 14.92 | 3.20 | 5.30 | 0.660 | < 0.001 | < 0.001 | 0.006 |
| Lactate ^2,^ mM | 0 | 0 | 0 | 0.26 | 0 | 0 | 9.711 | 0.997 | 0.988 | 0.006 |
| VFA concentration, mM | 70.8 | 94.8 | 93.2 | 106.9 | 82.0 | 102.1 | 2.11 | < 0.001 | < 0.001 | 0.62 |
| Molar percentage of individual VFA, mol/100 mol | | | | | | | | | | |
| Acetate | 61.0 | 57.9 | 57.1 | 57.9 | 58.9 | 58.5 | 0.21 | 0.002 | < 0.001 | < 0.001 |
| Propionate | 18.7 | 19.2 | 22.2 | 19.9 | 20.1 | 19.5 | 0.28 | 0.16 | 0.005 | 0.18 |
| Butyrate | 14.8 | 17.0 | 16.4 | 17.1 | 16.6 | 17.2 | 0.23 | 0.007 | 0.17 | 0.20 |
| Others | 5.5 | 5.8 | 4.4 | 5.1 | 4.4 | 4.8 | 0.11 | 0.008 | < 0.001 | 0.75 |
| Acetate to propionate ratio | 3.28 | 3.07 | 2.58 | 2.99 | 2.94 | 3.03 | 0.049 | 0.33 | 0.002 | 0.049 |
| **Third** **period** | | | | | | | | | | |
| pH | 7.14 | 7.20 | 7.01 | 6.84 | 7.08 | 7.00 | 0.032 | 0.024 | < 0.001 | 0.13 |
| Headspace gas |  |  |  |  |  |  |  |  |  |  |
| Hydrogen, μM | 56.5 | 42.3 | 54.3 | 41.4 | 48.8 | 38.7 | 2.61 | 0.003 | 0.03 | 0.60 |
| Methane, M | 18.9 | 14.2 | 18.0 | 13.8 | 16.4 | 13.1 | 0.87 | 0.003 | 0.04 | 0.58 |
| Dissolved hydrogen, μM | 10.3 | 18.2 | 12.7 | 34.2 | 14.6 | 28.2 | 1.29 | < 0.001 | < 0.001 | 0.005 |
| Lactate, mM^1^ | 0 | 0.09 | 0.35 | 2.10 | 0 | 7.77 | 0.124 | < 0.001 | < 0.001 | < 0.001 |
| VFA concentration, mM | 75.2 | 62.8 | 95.6 | 82.7 | 81.8 | 74.1 | 1.87 | 0.001 | < 0.001 | 0.74 |
| Molar percentage of individual VFA, mol/100 mol | | | | | | | | | | |
| Acetate | 60.7 | 53.3 | 56.6 | 52.5 | 58.2 | 51.6 | 0.52 | < 0.001 | 0.02 | 0.12 |
| Propionate | 21.8 | 22.2 | 24.6 | 25.3 | 23.5 | 24.9 | 0.34 | 0.19 | 0.001 | 0.78 |
| Butyrate | 12.6 | 14.0 | 14.3 | 13.4 | 13.9 | 14.7 | 0.37 | 0.57 | 0.58 | 0.39 |
| Others | 5.0 | 10.5 | 4.5 | 8.9 | 4.4 | 8.8 | 0.33 | < 0.001 | 0.009 | 0.23 |
| Acetate to propionate ratio | 2.79 | 2.45 | 2.30 | 2.13 | 2.48 | 2.10 | 0.046 | < 0.001 | < 0.001 | 0.45 |

VFA, volatile fatty acid

^1^ Fiber-rich diet was the same for three periods, while starch-rich diets were formulated by gradually replacing 1/3 of corn stover silage with maize meal. Starch-rich diets of period 1, 2, and 3 contained 50%, 70% and 90% concentrate respectively, and each period was last for 100 d.

^2^ The limit of detection (LOD) was 0.03 mM, the limit of quantitation (LOQ) was 0.10 mM.

**Table S****5. Comparison of nutrient digestibility of three periods between fiber-rich and starch-rich treatments (**n = 12/group).

| Item | Diets | | SEM | *P* |
| --- | --- | --- | --- | --- |
|  | Fiber-rich | Starch-rich |  |  |
| First period ^1^, g/kg | | | |  |
| DM | 547 | 597 | 10.1 | 0.002 |
| NDF | 507 | 506 | 14.8 | 0.97 |
| Starch | 878 | 958 | 2.3 | < 0.001 |
| Second period ^1^, g/kg | | | |  |
| DM | 535 | 654 | 10.3 | < 0.001 |
| NDF | 505 | 468 | 14.0 | 0.08 |
| Starch | 975 | 995 | 0.5 | < 0.001 |
| Third period ^1^, g/kg | | | |  |
| DM | 552 | 720 | 13.0 | < 0.001 |
| NDF | 507 | 434 | 25.8 | 0.07 |
| Starch | 978 | 997 | 0.4 | < 0.001 |

DM, dry matter; NDF, neutral detergent fiber

^1^ Fiber-rich diet was the same for three periods, while starch-rich diets were formulated by gradually replacing 1/3 of corn stover silage with maize meal. Starch-rich diets of period 1, 2 and 3 contained 50%, 70%, and 90% concentrate respectively, and each period was last for 100 d.

**Supplementary Text**

**Supplemental Materials and Methods –**

**Nutrient digestibility**

Feed, refusals, and fecal samples were collected daily from d 93 to 98 of each period. Daily samples of feed and refusals samples were collected and then mixed into a pooled sample for each period. Total feces were collected daily, weighed and mixed into pooled samples, and a subsample (1%) was frozen immediately at -20°C. All the samples were dried at 65°C for 48 h in a forced-air oven, and ground through a 1-mm screen for subsequent analysis of chemical composition. Nutrient digestibility was calculated based on nutrient intake and fecal output.

**Rumen samples collection**

Rumen samples were collected at 0, 2.5, and 6 h after the morning feeding on two consecutive days on d 99 to 100 of each period. About 500 mL of rumen content was collected by stainless-steel stomach tube with a rumen vacuum sampler, and the first 150 mL of rumen contents were discarded to avoid saliva contamination [1]. About 20 mL of sampled rumen content were used for immediately measuring pH with a portable pH meter (Starter 300; Ohaus Instruments Co. Ltd., Shanghai, China). Three 50-mL subsamples were immediately frozen in liquid N_2_ and stored at -80°C for DNA extraction. Two 35-mL subsamples were immediately transferred to 50-mL plastic syringes for measuring dissolved hydrogen concentrations [2]. Three 5-mL subsamples of rumen contents were collected and centrifuged at 12,000 × g for 10 min at 4°C. A 1.5 mL aliquot of supernatant was acidified with 0.15 mL of meta-phosphoric acid (25%, w/v), and stored at -20 °C for subsequent measurement of fermentation products.

All the cattle were weighed and slaughtered by bleeding of the carotid artery at the end of the experiment after fasting for 12 h. After slaughter, the rumen was separated and opened, and pH was immediately measured in rumen contents with a portable pH meter. Then, the rumen was emptied and weighted. Four segments of epithelial tissue (5 × 5 cm) from the ventral sac of the rumen were excised from the muscular and serosal layers by blunt dissection, and immediately washed in cold phosphate buffered saline (PBS) until the PBS was clear. The first epithelial sample was immediately snap-frozen in liquid nitrogen for tissue RNA extraction. One ruminal epithelial sample was fixed in 4% paraformaldehyde solution (PFA) (Sigma, USA) for histomorphometric microscopy analysis. Two ruminal epithelial samples were immediately fixed in cold PBS to measure papillae size. The site of sampling was kept constant for all the animals.

***In-vitro* ruminal fermentation**

The present study included two *in vitro* experiments with ruminal batch cultures with rumen inoculum from the final experimental period. The *in vitro* experiment 1 compared two treatments, fiber-rich substrate (Supplemental Table S1) inoculated with rumen fluid from animals fed the fiber-rich diet, and starch-rich substrate (Supplemental Table S1) inoculated with rumen fluid from animals fed the starch-rich diet. The *in vitro* experiment 2 used rice straw as incubation substrate inoculated with rumen fluid from animals fed the fiber-rich or the starch-rich diet.

*In vitro* ruminal fermentation was performed according to the procedure of Wang et al., (2016) [3]. Briefly, rumen contents were collected before the morning feeding using a stainless-steel stomach tube, filtered through five layers of cheesecloth, and then mixed with pre-wed McDougall's buffer (volume ratio of 1 to 4) to prepare the buffered rumen fluid for subsequent *in vitro* ruminal inoculation. Buffered rumen fluid (60 mL) was delivered into 135-ml bottles containing 1g of substrate. All of these procedures were performed under a stream of CO_2_. Bottles were sealed and incubated at 39.5 ℃ for 48 h. Total gas volume was measured at 48 h using the method described by Wang et al. [4], and gas samples were collected in evacuated tubes for subsequent measurement of methane concentration. A 2-mL sample from the liquid phase was collected from each bottle for the analyses of fermentation end products. Solid residues were filtered into pre-weighed Gooch filter crucibles and dried at 105 °C for 24 h, and weighed to determine degradation of incubated substrates.

**Sample analyses**

Contents of DM (method 945.15), OM (method 942.05) and CP (method 945.01, total N ×6.25) were analyzed using the methods of Association of Official Analytical Chemists [5]. Neutral detergent fiber and acid detergent fiber contents were determined with inclusion of a heat stable *α*-amylase, and expressed with residual ash [6]. Starch content was measured using amyloglucosidase [7]. Headspace gas was collected by rumenocentesis according to Moate et al. [8]. The gas cap of the central rumen was punctured with a 150-mm long, 14-gauge needle after shearing and disinfection (75% ethanol) of the skin. A 50-mL syringe was attached to the needle to collect 30 mL of headspace gas from the rumen. Dissolved gases were extracted from the liquid phase of rumen contents into the gas phase [2], measured using a gas chromatograph (Agilent 7890A, Agilent Inc., Palo Alto, CA) [1]. Individual volatile fatty acids (VFA) concentrations were analyzed by gas chromatography (Agilent 7890A, Agilent Inc., Palo Alto, CA) [2]. The concentration of lactate was analyzed by HPLC (Agilent LC1290, Agilent Inc., Palo Alto, CA) [9].

**Microbial DNA extraction**

Microbial DNA was extracted using the modified protocol by Ma et al. [10], and purified with phenol/chloroform/isopentyl alcohol (25:24:1 vol/vol/vol, Solarbio Co., Shanghai, China) [11]. The vacuum-dried DNA pellets were dissolved in Tris EDTA buffer (Tris 10 mM, EDTA 1 mM, pH = 8.0) after being washed with 70% ethanol alcohol twice. The concentration and purity of DNA were measured using an ND-2000 spectrophotometer (NanoDrop Technologies, Wilmington, DE). All DNA samples were stored at −80 °C until subsequent analyses.

**16S ribosomal DNA sequencing and analysis**

The V3 and V4 region was amplified with a 6 bp barcode unique to each sample by using the universal primers (341F: 5′-CCTAYGGGRBGCASCAG-3′, 806R: 5′-GGACTACNNGGGTATCTAAT-3′) [12]. After PCR amplification, all amplicon libraries were sequenced on MiSeq platform (Illumina, San Diego, CA, USA) at Shanghai Biozeron Biological Technology Co. Ltd, and the barcodes and sequencing primers were removed before data processing.

For 16S data, passed sequences were dereplicated and subjected to the DADA2 algorithm to identify indel-mutations and substitutions [13], which resolves amplicon sequence errors to generate amplicon sequence variants (ASVs). The phylogenetic affiliation of each 16S rRNA gene sequence was analyzed by RDP Classifier (http://rdp.cme.msu.edu/) against the SILVA (SSU138) 16S rRNA database using a confidence threshold of 70% [14]. Beta diversity was performed using the *vegan* package [15] in R based on unweighted UniFrac dissimilarity matrix. Analysis of similarities (ADONIS) was performed to indicate group similarity, and the *p* values were determined based on 999 permutations. The Spearman’s correlation coefficients (*r*) between the microbial genera were harvested using Hmic R package, and only those in accordance with | *r* |>0.5 and *p* <0.05 were used in the following analysis. The software Gephi [16] (version 0.9.2, https://gephi.org/) was used to visualize the following correlation network. Random forest (RF) classification was performed with the group as the class using R randomForest package v4.6–14 [17]. The number of trees (ntree) in the forest was set to 500 and the number of features randomly sampled for each split of the tree (m_try_) was 14. The out-of-bag decrease in accuracy was averaged in all trees for a variable (mean decrease accuracy) and used as the measurement of variable importance.

**Shotgun metagenome sequencing**

For each sample, 1 μg of genomic DNA was sheared by Covaris S220 Focused-ultrasonicator (Woburn, MA USA) and sequencing libraries were prepared with a fragment length of approximately 350 bp (ranging from 300 to 400 bp). All samples were sequenced on the HiSeq X platform (Illumina, San Diego, CA, USA) with pair-end 150 bp (PE150) mode. After sequencing, low-quality reads, contaminated adaptors and host reads (Bos_taurus. UMD3.1 [18] and hg19 [19]) were removed from the raw sequenced reads by Trimmomatic (http://www.usadellab.org/cms/?page=trimmomatic) and BWA package (version 0.7.12), respectively [20]. Subsequently, the clean data reads were used as input for MEGAHIT (version 1.1.1) [21] with “--min-contig-len 500” parameters. Prodigal (version 2.6.3) [22] was used to predict the contigs from each sample with ‘-meta’ parameters, and the ORFs derived from assembled contigs were maintained and clustered into a nonredundant data set by CD-HIT ((version 4.6.7v.4.8.1, parameter: -n 9 -g 1 -c 0.95 -G 0 -M 0 -d 0 -aS 0.9) [23], thus a 12.0 Gb pan-metagenome was constructed based on the assembled contigs with an average N50 length of 1.7 Kb, including 17.9 million non-redundant genes, and the average length of open read frame was 631 bp. Salmon software [24] was applied to calculate the gene relative abundance by using following equation:

$$Relative abundance= \frac{N_{g}}{Lg}\times\frac{1}{\sum j\frac{N_{j}}{L_{j}}}\times{10}^{6}$$

where *Ng* is the read count, i.e., the average number of reads mapped to the *g* gene; and *Lg* is the gene length, i.e., the number of nucleotides in the *g* gene. The index *j* stands for the set of all genes determined in a catalog, and *g* is an index indicating a particular gene.

**Functional annotation and taxonomic assignment**

Taxonomy of clean reads for each sample was determined by Kraken [25] using the MiniKraken database (2020.03, ftp://ftp.ccb.jhu.edu/pub/data/kraken2_dbs/). All reads were classified into seven phylogenetic levels (domain, phylum, class, order, family, genus, species) or unclassified. The abundances of taxonomy clades were estimated using Bracken (https://ccb.jhu.edu/software/bracken/) which can produce accurate species- and genus-level abundance even in multiple near-identical species. Relative abundance of certain level is a total of abundance of species belonging to certain level.

The functional annotation of gene sets was performed with KofamKOALA [26] (https://www.genome.jp/tools/kofamkoala/). The gene set was aligned with the CAZy database [27] using HMMER [28] and corresponding annotations for CAZyme were harvested. Hydrogenases (e.g., NiFe-, FeFe-, and Fe-hydrogenases) were identified with HydDB by DIAMOND [29] with an e-value threshold of 1e-50, one maximum target sequence per query, and results were then filtered (length of amino acid > 40 residues, sequence identity > 60%). Terminal reductases were identified by referring to Greening et al [30]. Specifically, genes encoding for subunits of terminal reductases (including *acsB*, *aprA*, *asrA*, *cydA*, *dmsA*, *dsrA*, *frdA*, *hydB*, *mcrA*, *napA*, *narG*, *nifH*, and *nrfA*) were identified by DIAMOND searches against respective gene sequences (with an e-value threshold of 1e-50, coverage values exceeding 90% and identity values exceeding 50 or 60%). All genes were subjected to taxonomic assignment using DIAMOND based on BLASTP searches against the NCBI-NR (October 2018; approximately 550 M sequences).

**Recruitment to the microbial genomes**

The sequences of all genomes of cultured rumen bacteria from Hungate1000 collection [31] were retrieved form the Joint Genome Institute (JGI) genome partial. The abundance of each genome was calculated by metawrap quant_bins module with default parameters [32]. Then the genomic ranks of attributes were evaluated by Correlation, ReliefF, Symmetrical Uncert, and multi-cluster feature selection (MCFS) methods using Waikato Environment for Knowledge Analysis (WEKA) (version 3.8.4, Hamilton, New Zealand) [33]. RobustRankAggreg (RRA) R package [34] was selected for comprehensive analysis.

**qPCR analysis of genes in ruminal epithelium and targeted bacterial species**

Total RNA of the ruminal epithelium was extracted from the tissue using TRIzol® Reagent according the manufacturer’s instructions (Invitrogen) and genomic DNA was removed using DNase I (Takara). The RNA quality was then determined using 2100 Bioanalyser (Agilent) and quantified using the ND-2000 (NanoDrop Technologies). Total RNA was used for reverse transcription using a PrimeScript® RT reagent kit with gDNA Eraser (Takara Bio, Otsu, Japan). Quantitative real time PCR (q-PCR) was performed to determine gene expression using SYBR® Premix Ex Taq™ II (Takara, Dalian, China) and LightCycler 480 II Instrument (Roche, Basel, Switzerland). Relative expression of all target genes was determined and normalized to the reference genes (*GAPDH* and *β-actin*) using the 2^–ΔΔCt^ method [35]. Primers were synthesized by Sangon Biotech Co.; Ltd. (Shanghai, China; Supplemental Table S2).

Quantification of *Fibrobacter succingenes* and *Ruminobacter amylophilus* was conducted by qPCR with primers validated in our laboratory (Supplemental Table S3) according to the procedures described by Ma et al. [36]. Copies of 16S rRNA gene of target species were estimated by relating the cycle threshold value to standard curves and expressed as copies per milliliter of rumen fluid (log_10_ transformed).

**References**

1. Wang M, Wang R, Janssen PH, Zhang XM, Sun XZ, Pacheco D, et al. Sampling procedure for the measurement of dissolved hydrogen and volatile fatty acids in the rumen of dairy cows. J Anim Sci. 2016; 94:1159-1169.

2. Wang M, Sun XZ, Janssen PH, Tang SX, Tan ZL. Responses of methane production and fermentation pathways to the increased dissolved hydrogen concentration generated by eight substrates in in vitro ruminal cultures. Anim Feed Sci Technol. 2014; 194:1-11.

3. Wang M, Wang R, Tang SX, Tan ZL, Zhou CS, Han XF, et al. Comparisons of manual and automated incubation systems: Effects of venting procedures on in vitro ruminal fermentation. Livest Sci. 2016; 184:41-45.

4. Wang M, Janssen PH, Sun XZ, Muetzel S, Tavendale M, Tan ZL, et al. A mathematical model to describe in vitro kinetics of H_2_ gas accumulation. Anim Feed Sci Technol. 2013; 184:1-16.

5. AOAC, Horwitz W. Official methods of analysis of AOAC International, 16th ed. Association of Official Analytical Chemists, Arlington, VA, 1995.

6. Soest PJ, Robertson JB, Lewis BA. Symposium: Carbohydrate methodology, metabolism, and nutritional implications in dairy cattle. J Dairy Sci. 1991; 74.

7. Kartchner RJ, Theurer B. Comparison of hydrolysis methods used in feed, digesta, and fecal starch analysis. J Agric Food Chem 1981; 29:8-11.

8. Moate PJ, Clarke T, Davis LH, Laby RH. Rumen gases and bloat in grazing dairy cows. J Agric Sci. 1997; 129:459-469.

9. Canale A, Valente ME, Ciotti A. Determination of volatile carboxylic acids (C1–C5i) and lactic acid in aqueous acid extracts of silage by high performance liquid chromatography. J Sci Food Agric. 1984; 35:1178-1182.

10. Ma ZY, Zhang XM, Wang R, Wang M, Liu T, Tan ZL. Effects of chemical and mechanical lysis on microbial DNA yield, integrity, and downstream amplicon sequencing of rumen bacteria and protozoa. Front Microbiol. 2020;11:581227.

11. Minas K, McEwan NR, Newbold CJ, Scott KP. Optimization of a high-throughput CTAB-based protocol for the extraction of qPCR-grade DNA from rumen fluid, plant and bacterial pure cultures. Fems Microbiol Lett. 2011; 325:162-169.

12. Zakrzewski M, Goesmann A, Jaenicke S, Junemann S, Eikmeyer F, Szczepanowski R, et al. Profiling of the metabolically active community from a production-scale biogas plant by means of high-throughput metatranscriptome sequencing. J Biotechnol. 2012; 158:248-258.

13. Callahan BJ, McMurdie PJ, Rosen MJ, Han AW, Johnson AJ, Holmes SP. DADA2: High-resolution sample inference from Illumina amplicon data. Nat Methods. 2016; 13:581-583.

14. Amato KR, Yeoman CJ, Kent A, Righini N, Carbonero F, Estrada A, et al. Habitat degradation impacts black howler monkey (*Alouatta pigra*) gastrointestinal microbiomes. ISME J. 2013; 7:1344-1353.

15. Dixon P. VEGAN, a package of R functions for community ecology. J Veg Sci. 2003; 14:927-930.

16. Bastian M, Heymann S, Jacomy M. Gephi: an open source software for exploring and manipulating networks. In third international ICWSM conference. 2009; 361-362.

17. Liaw A, Wiener M. Classification and regression by RandomForest. R news. 2002; 2:18-22.

18. Shamimuzzaman M, Le Tourneau JJ, Unni DR, Diesh CM, Triant DA, Walsh AT, et al. Bovine genome database: new annotation tools for a new reference genome. Nucleic Acids Res. 2019; 48:D676-D681.

19. Collins, F. S., Lander, E. S., Rogers, J., Waterston, R. H., & Conso, I. H. G. S. Finishing the euchromatic sequence of the human genome. Nature. 2004; 431:931-945.

20. Li H, Durbin R. Fast and accurate long-read alignment with burrows-wheeler transform. Bioinformatics. 2010; 26:589-595.

21. Li D, Liu CM, Luo R, Sadakane K, Lam TW. MEGAHIT: an ultra-fast single-node solution for large and complex metagenomics assembly via succinct de Bruijn graph. Bioinformatics. 2015; 31:1674-1676.

22. Hyatt D, Chen GL, LoCascio PF, Land ML, Larimer FW, Hauser LJ. Prodigal: prokaryotic gene recognition and translation initiation site identification. BMC Bioinformatics. 2010; 11.

23. Fu L, Niu B, Zhu Z, Wu S, Li W. CD-HIT: accelerated for clustering the next-generation sequencing data. Bioinformatics. 2012; 28:3150-3152.

24. Patro R, Duggal G, Love MI, Irizarry RA, Kingsford C. Salmon provides fast and bias-aware quantification of transcript expression. Nat Methods. 2017; 14:417-419.

25. Wood DE, Salzberg SL. Kraken: ultrafast metagenomic sequence classification using exact alignments. Genome Biol. 2014; 15.

26. Aramaki T, Blanc-Mathieu R, Endo H, Ohkubo K, Kanehisa M, Goto S, et al. KofamKOALA: KEGG ortholog assignment based on profile HMM and adaptive score threshold. Bioinformatics. 2019; 36:2251-2252.

27. Lombard V, Ramulu HG, Drula E, Coutinho PM, Henrissat B. The carbohydrate-active enzymes database (CAZy) in 2013. Nucleic Acids Res. 2014; 42:D490-D495.

28. Wheeler TJ, Eddy SR. nhmmer: DNA homology search with profile HMMs. Bioinformatics. 2013; 29:2487-2489.

29. Buchfink B, Xie C, Huson DH. Fast and sensitive protein alignment using DIAMOND. Nat Methods. 2015; 12:59-60.

30. Greening C, Geier R, Wang C, Woods LC, Morales SE, McDonald MJ, et al. Diverse hydrogen production and consumption pathways influence methane production in ruminants. ISME J. 2019; 13:2617-2632.

31. Seshadri R, Leahy SC, Attwood GT, Teh KH, Lambie SC, Cookson AL, et al. Cultivation and sequencing of rumen microbiome members from the Hungate1000 Collection. Nat Biotechnol. 2018; 36:359-367.

32. Uritskiy GV, DiRuggiero J, Taylor J. MetaWRAP-a flexible pipeline for genome-resolved metagenomic data analysis. Microbiome. 2018; 6.

33. Witten IH, Frank E, Hall MA, Pal CJ. Data mining: practical machine learning tools and techniques. 4th ed. Morgan Kaufmann, 2016.

34. Kolde R, Laur S, Adler P, Vilo J. Robust rank aggregation for gene list integration and meta-analysis. Bioinformatics. 2012 ;28:573-580.

35. Schmittgen TD, Livak KJ. Analyzing real-time PCR data by the comparative CT method. Nat Protoc. 2008; 3:1101-1108.

36. Ma Z, Wang R, Wang M, Zhang X, Mao H, Tan Z. Short communication: variability in fermentation end-products and methanogen communities in different rumen sites of dairy cows. J Dairy Sci. 2018; 101:5153-5158.
